# LRRK1 functions as a scaffold for PTP1B-mediated EGFR sorting into ILVs at the ER–endosome contact site

**DOI:** 10.1242/jcs.260566

**Published:** 2023-03-02

**Authors:** Hiroshi Hanafusa, Keitaro Fujita, Misa Kamio, Shiori Iida, Yasushi Tamura, Naoki Hisamoto, Kunihiro Matsumoto

**Affiliations:** ^1^ Division of Biological Science, Graduate School of Science, Nagoya University, Chikusa-ku, Nagoya 464-8602, Japan; ^2^ Department of Chemistry, Faculty of Science, Yamagata University, Shirakawa, Yamagata 990-8560, Japan

**Keywords:** EGFR, LRRK1, PTP1B, Intraluminal vesicles, Endosome, Membrane contact sites

## Abstract

Proper control of epidermal growth factor receptor (EGFR) signaling is important for maintaining cellular homeostasis. Given that EGFR signaling occurs at the plasma membrane and endosomes following internalization, endosomal trafficking of EGFR spatiotemporally regulates EGFR signaling. In this process, leucine-rich repeat kinase 1 (LRRK1) has multiple roles in kinase activity-dependent transport of EGFR-containing endosomes and kinase-independent sorting of EGFR into the intraluminal vesicles (ILVs) of multivesicular bodies. Active, phosphorylated EGFR inactivates the LRRK1 kinase activity by phosphorylating Y944. In this study, we demonstrate that LRRK1 facilitates EGFR dephosphorylation by PTP1B (also known as PTPN1), an endoplasmic reticulum (ER)-localized protein tyrosine phosphatase, at the ER–endosome contact site, after which EGFR is sorted into the ILVs of endosomes. LRRK1 is required for the PTP1B–EGFR interaction in response to EGF stimulation, resulting in the downregulation of EGFR signaling. Furthermore, PTP1B activates LRRK1 by dephosphorylating pY944 on the contact site, which promotes the transport of EGFR-containing endosomes to the perinuclear region. These findings provide evidence that the ER–endosome contact site functions as a hub for LRRK1-dependent signaling that regulates EGFR trafficking.

## Teaser

**Summary:** LRRK1 facilitates dephosphorylation of EGFR by PTP1B, an ER-localized protein tyrosine phosphatase, at the ER–endosome contact site. This is required for EGFR sorting into ILVs and downregulation of EGFR signaling.

## INTRODUCTION

Epidermal growth factor receptor (EGFR) signaling regulates a variety of responses including cell growth, proliferation, and motility (Schlessinger, 2000). Given that excessive EGFR signaling can result in the development and progression of various types of cancer, its regulation is important for maintaining cellular homeostasis (Sigismund et al., 2018; Yarden and Pines, 2012). The binding of EGF ligands to EGFR on the cell surface activates downstream signaling pathways. Activation of EGFR also initiates the events leading to its endocytosis. Internalized EGFR is associated with early endosomes, where it is either returned to the cell surface or sorted into lysosomes for degradation. EGFR destined for degradation is transported by the dynein motor protein along microtubules into lysosomes via multivesicular bodies (MVBs)/late endosomes. During this process, EGFR is incorporated into intraluminal vesicles (ILVs) of MVBs, which results in the physical removal of the catalytic domain of EGFR from the cytoplasm and effectively terminates downstream signaling before it can be degraded by lysosomes. EGFR signaling occurs not only at the plasma membrane but also at endosomes following internalization (Irannejad et al., 2015; Miaczynska et al., 2004; Vieira et al., 1996). Therefore, the endosomal trafficking of EGFR is essential for determining the amplitude and duration of EGFR signaling.

Ligand-dependent dimerization and autophosphorylation of receptor tyrosine kinases is a ubiquitous signaling mechanism (Lemmon and Schlessinger, 2010). Given that tyrosine phosphorylation is reversible, protein tyrosine phosphatases (PTPs) contribute to the regulation of activated receptors (Tonks, 2006). In particular, PTP1B (also known as PTPN1) has been implicated in the direct dephosphorylation and negative regulation of EGFR (Flint et al., 1997; Haj et al., 2003); however, it is evident that the actual role of PTP1B is much more complex. PTP1B localizes to the cytoplasmic face of the endoplasmic reticulum (ER) (Frangioni et al., 1992). Recent studies have revealed that the membrane contact site (MCS) between the ER and the endosome provides a site in which PTP1B interacts with endocytosed EGFR (Eden et al., 2010; Haj et al., 2002). This interaction is likely short-lived and stabilized by additional factors, such as annexin A1, a key regulator of ER–endosome contact (Eden et al., 2016). Given that PTP1B-mediated dephosphorylation of EGFR appears to be important for the efficient sorting of EGFR into the ILVs of endosomes, PTP1B activity suppresses EGFR signaling by dephosphorylating EGFR and promoting its sorting into ILVs (Stuible and Tremblay, 2010; Wong et al., 2018). However, the detailed mechanism underlying EGFR sorting remains unclear.

We previously reported that leucine-rich repeat kinase 1 (LRRK1) is required for the uptake of EGFR into the endosomal lumen (Hanafusa et al., 2011). LRRK1 is related to Park8 (also known as LRRK2), which is encoded by a gene associated with familial Parkinson's disease (Bosgraaf and Van Haastert, 2003). It belongs to the ROCO family of proteins, members of which contain the Ras of complex protein (ROC) GTPase domain, the MAPKKK-like kinase domain, and several protein–protein interaction domains (Bosgraaf and Van Haastert, 2003). Following stimulation with EGF, LRRK1 forms a complex with EGFR, and the LRRK1–EGFR complex is subsequently endocytosed into early endosomes, where LRRK1 regulates the transport of EGFR from the early endosomes to lysosomes via the MVBs/late endosomes in a manner dependent upon its kinase activity (Hanafusa et al., 2011, 2019; Ishikawa et al., 2012; Kedashiro et al., 2015). LRRK1 also facilitates the sorting of EGFR into the ILVs of the MVBs, independently of its kinase activity (Hanafusa et al., 2011).

In the present study, we demonstrate that LRRK1 is necessary for endosomal EGFR dephosphorylation by ER-localized PTP1B at the ER–endosome contact site and for sorting EGFR into ILVs. As LRRK1 is essential for the PTP1B–EGFR interaction in response to EGF stimulation, our findings suggest that LRRK1 serves as a scaffold protein at the ER–endosome contact site to promote EGFR dephosphorylation by PTP1B and thereby downregulate EGFR signaling.

## RESULTS

### LRRK1 is required for EGFR dephosphorylation and sorting into ILVs

**Fig. 1.**
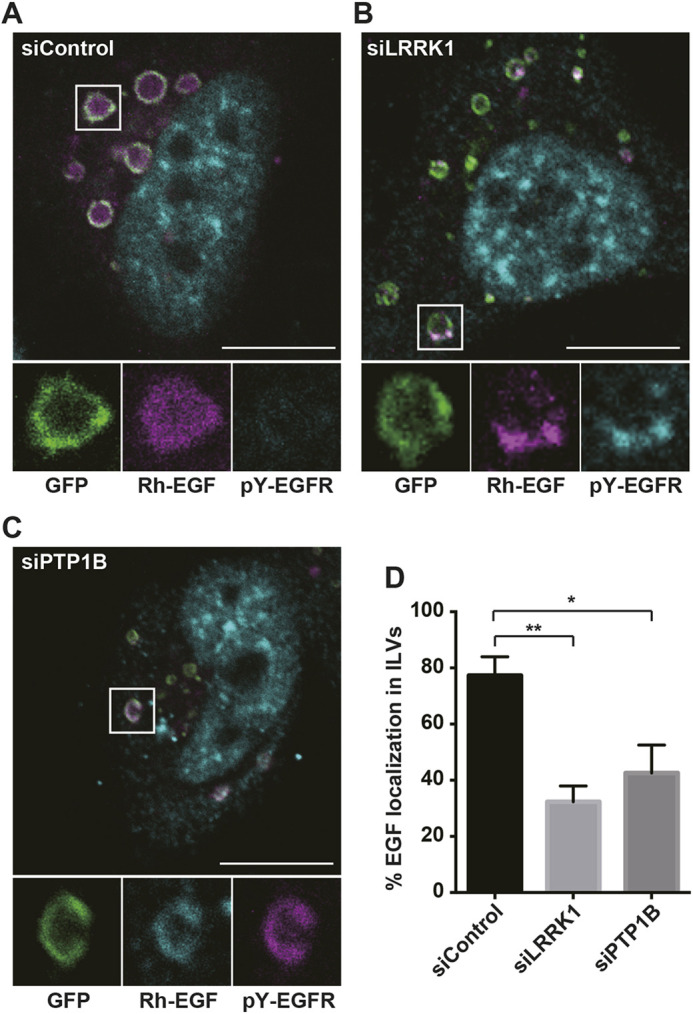
**LRRK1 and PTP1B are required for EGFR dephosphorylation and sorting into ILVs.** (A–C) Effects of LRRK1 or PTP1B depletion on EGFR sorting into ILVs and the activation state of EGFR localized to the endosomes. HeLa S3 cells treated with control siRNA (A), LRRK1-specific siRNA (B), or PTP1B-specific siRNA (C) were transfected with GFP–Rab5(Q79L). After 16 h of serum starvation, the cells were stimulated with Rh–EGF (50 ng/ml) for 30 min, fixed and immunostained with anti-pY1068-EGFR antibodies (cyan). Images were captured by confocal microscopy. Confocal images from the boxed region are magnified to show individual enlarged endosomes. Scale bars: 10 µm. (D) Quantification of Rh–EGF localization into the endosomal lumen. Data are presented as the percentage of Rh–EGF localized into the ILVs of the endosomes out of the total number of endosomes (diameter; >1 µm). Values reflect the mean±s.d. of three independent experiments with an average of 30 cells (>100 endosomes) scored per experiment. *P<0.05; **P<0.01 (Dunnett's multiple-comparison test).

EGFR localized in clathrin-coated microdomains on endosomes is sorted into ILVs within MVBs and delivered to lysosomes for degradation (Raiborg et al., 2002, 2006). Our previous studies have indicated that LRRK1 regulates EGFR sorting into ILVs (Hanafusa et al., 2011). To determine the regulatory mechanism, HeLa S3 cells were transfected with a constitutively active Rab5 expression vector, GFP-tagged Rab5(Q79L), to increase the rate of endosome fusion (Stenmark et al., 1994). Increased Rab5 activity results in the formation of enlarged endosomes, in which ILV sorting can be readily observed by confocal immunofluorescence microscopy (Raiborg et al., 2002, 2006). EGFR localization was assessed using fluorescently labeled rhodamine-conjugated EGF (Rh–EGF). As observed previously (Hanafusa et al., 2011), when control siRNA (siControl)-transfected cells were stimulated for 30 min with Rh–EGF, the majority of the Rh–EGF was translocated from the membrane to the ILVs in Rab5(Q79L)-induced enlarged endosomes (Fig. 1A,D). In contrast, in LRRK1-knockdown (siLRRK1) cells (Fig. S1A), a large portion of the Rh–EGF was retained on the membrane of endosomes and its uptake into the lumen was inhibited (Fig. 1B,D). Quantification of EGF labeling revealed that ∼80% of the labeling was localized to the inner membrane of control cells, whereas in cells treated with LRRK1 siRNA, localization was only 30% (Fig. 1D). Another siRNA oligonucleotide against LRRK1 (LRRK1 siRNA a#1) also inhibited Rh–EGF localization to ILVs (Fig. S2). These results indicate that LRRK1 is required for EGFR sorting to ILVs. Moreover, we previously demonstrated that LRRK1 kinase activity is not essential for this step (Hanafusa et al., 2011). Thus, LRRK1 functions as a scaffold in this process, which is independent of its kinase activity.

EGF binding activates the intrinsic kinase activity of EGFR, resulting in the autophosphorylation of multiple receptor tyrosine residues (Lemmon and Schlessinger, 2010; Schlessinger, 2000). Given that the uptake of EGFR into the endosomal lumen is important for downregulating EGFR signaling, we determined the effect of LRRK1 depletion on the activation state of EGFR localized in the enlarged endosomes using an antibody against Y-1068-phosphorylated EGFR (pY-EGFR) that specifically recognizes activated EGFR (Downward et al., 1984). Immunostaining with anti-pY1068-EGFR antibodies revealed that in control siRNA-treated cells, EGFRs incorporated into ILVs were not phosphorylated (Fig. 1A). These results are consistent with previous reports that unphosphorylated EGFRs on the endosomal membrane are subsequently sorted into the endosomal lumen (Eden et al., 2010; Wong et al., 2018). In cells treated with LRRK1 siRNA, the EGFR accumulated on the endosomal membrane was phosphorylated (Fig. 1B). Thus, LRRK1 knockdown results in the accumulation of active EGFR on the membrane of the endosomes. These results suggest that tyrosine dephosphorylation is required for EGFR sorting into ILVs and this step is dependent upon LRRK1.

**Fig. 2.**
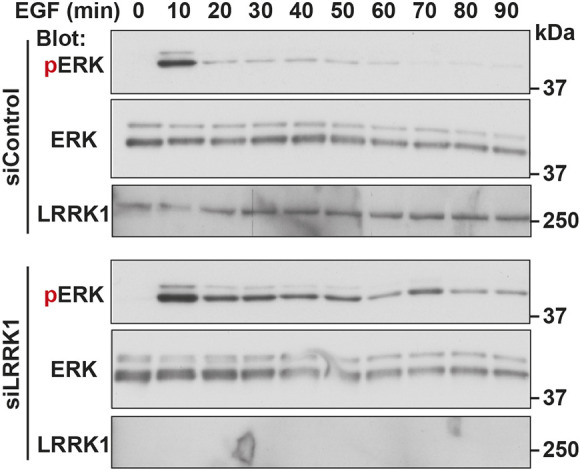
**Effect of LRRK1 depletion on ERK activation in response to EGF stimulation.** HeLa S3 cells were treated with control or LRRK1 siRNA. After 16 h of serum starvation, the cells were stimulated with EGF (10 ng/ml) for the indicated times. Cell lysates were prepared and immunoblotted (Blot) with the indicated antibodies. Blot is representative of three experimental repeats.

EGFR signaling occurs at the plasma membrane and endosomes following internalization (Irannejad et al., 2015; Miaczynska et al., 2004; Vieira et al., 1996). The treatment of cells with LRRK1 siRNA resulted in the accumulation of phosphorylated active EGFR on the endosomal membranes (Fig. 1B), raising the possibility that LRRK1 siRNA increases the activity of the downstream signaling pathways. To test this possibility, we monitored ERK (herein referring to ERK1 and ERK2, also known as MAPK3 and MAPK1, respectively) activation, a kinase downstream of EGFR signaling (Lemmon and Schlessinger, 2010; Yarden and Pines, 2012), by immunoblotting with a phospho-specific anti-ERK antibody. When HeLa S3 cells were stimulated with EGF, ERK activation was observed 10 min after stimulation, which then rapidly decreased (Fig. 2). In contrast, LRRK1 knockdown resulted in sustained ERK activation after EGF stimulation (Fig. 2). Consistent with this, EGFR phosphorylation persisted in LRRK1-depleted cells (Fig. S3). These results suggest that LRRK1 is required for downregulating EGFR signaling by promoting the dephosphorylation of EGFR on endosomal membranes and its sorting into ILVs.

### LRRK1 is required for PTP1B-mediated EGFR dephosphorylation and sorting into ILVs

Based on the above results, LRRK1 may induce tyrosine dephosphorylation of EGFR, which would provide a directional effect on EGFR incorporation into ILVs. Recent studies have demonstrated that endocytosed EGFR is dephosphorylated at the ER–endosome contact site by ER-localized PTP1B (Eden et al., 2010; Haj et al., 2002). Therefore, we examined the effect of PTP1B depletion on EGFR sorting into ILVs. We observed that depletion of PTP1B with siRNA (siPTP1B) (Fig. S1A) in Rab5(Q79L)-transfected HeLa S3 cells reduced the intraluminal transport of Rh–EGF into enlarged endosomes and resulted in the accumulation of Rh–EGF on the endosomal membrane (Fig. 1C,D). Furthermore, these Rh–EGF signals were labeled with anti-pY1068-EGFR antibodies as observed in LRRK1-knockdown cells (Fig. 1C). The results confirm that PTP1B is required for the dephosphorylation of EGFR on endosomal membranes as well as sorting into ILVs. In cells depleted of LRRK1 or PTP1B, endosomes appeared smaller than in the control and were distributed around the plasma membrane (Fig. 1A–C). These results suggest that depletion of LRRK1 or PTP1B might delay fusion between endosomes.

**Fig. 3.**
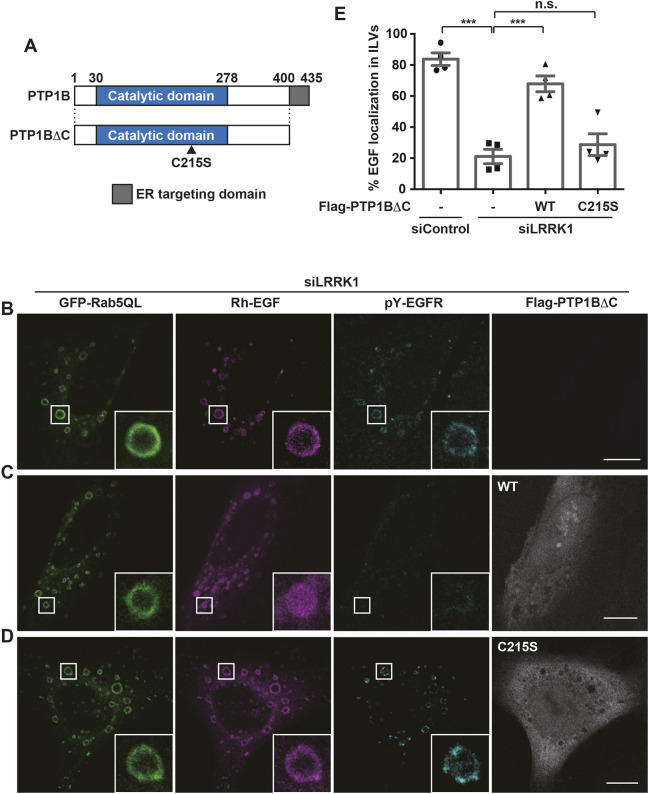
**LRRK1 is required for PTP1B-mediated EGFR dephosphorylation and sorting into ILVs.** (A) Schematic diagrams of the PTP1B protein. PTP1BΔC is shown below. The catalytically inactive C215S mutation is indicated by an arrowhead. (B–D) Effects of PTP1BΔC on EGFR sorting into ILVs and the activation state of EGFR localized to the endosomes in LRRK1 siRNA-treated cells. HeLa S3 cells treated with LRRK1 siRNA were co-transfected with GFP–Rab5(Q79L) and empty vector (B), Flag–PTP1BΔC(WT) (C) or the Flag–PTP1BΔC(C215S) mutant (D). After 16 h of serum starvation, the cells were stimulated with Rh–EGF (50 ng/ml) for 30 min, fixed and immunostained with anti-pY1068-EGFR (cyan) and anti-Flag antibodies (gray). Images were captured by confocal microscopy. The boxed regions are magnified in the insets. Scale bars: 10 µm. (E) Quantification of Rh–EGF localization to the lumen of the endosomes. Data are presented as the percentage of Rh–EGF localization into the ILVs of the endosomes out of the total number of endosomes (diameter; >1 µm). Values reflect the mean±s.d. of four independent experiments with an average of 30 cells (>100 endosomes) scored per experiment. ***P<0.001; n.s., not significant (Dunnett's multiple-comparison test).

Next, we examined the relationship between PTP1B and LRRK1 in regulating EGFR sorting into ILVs. Because LRRK1 knockdown induced the accumulation of phosphorylated EGFR on the endosomal membranes (Fig. 1B), it is likely that in the absence of LRRK1, ER-localized PTP1B could not sort EGFR into ILVs due to the defective dephosphorylation of EGFR on the endosomes. If so, overexpression of a cytoplasmic form of PTP1B in LRRK1-depleted cells would be expected to rescue the EGFR sorting defect. Previous studies have shown that PTP1B is anchored to the ER membrane at its C-terminus (Frangioni et al., 1992). Removal of the last 35 amino acids of the C-terminus (PTP1BΔC) would enable it to be distributed in the cytoplasm (Fig. 3A). When PTP1BΔC was overexpressed in LRRK1-depleted cells, EGFR dephosphorylation and sorting into ILVs were markedly enhanced (Fig. 3B,C,E). These effects were dependent upon the tyrosine phosphatase activity of PTP1B because the catalytically inactive PTP1BΔC(C215S) mutant (Fig. 3A) was unable to suppress LRRK1 siRNA defects (Fig. 3D,E). These results suggest that LRRK1 links PTP1B to the dephosphorylation of EGFR and its endosomal sorting.

### LRRK1 is dispensable for EGF-induced formation of ER–endosome contact sites

**Fig. 4.**
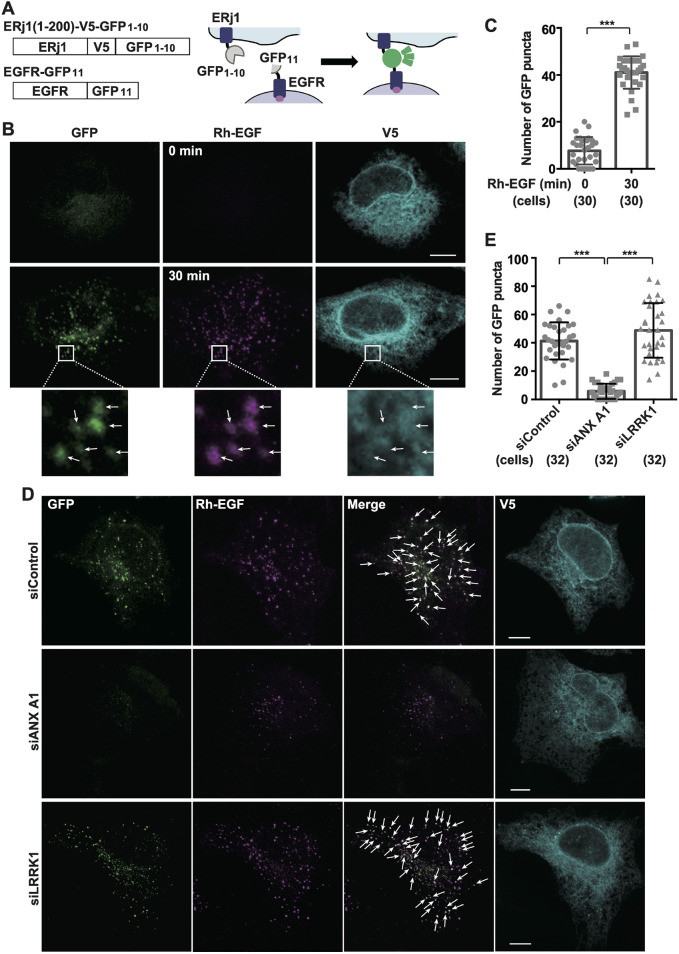
**Effect of LRRK1 depletion on EGF-induced ER–endosome contact site formation.** (A) Schematic diagrams of split-GFP fusion proteins. (B) Colocalization of assembled split-GFP fragments with Rh–EGF in ER. HeLa S3 cells were co-transfected with ERj1(1–200)–V5–GFP_1-10_ and EGFR–GFP_11_. After 16 h of serum starvation, the cells were stimulated with Rh–EGF (50 ng/ml) for 0 or 30 min, fixed and immunostained with anti-V5 antibodies (cyan). Images were captured by confocal microscopy. The boxed regions are magnified to show GFP puncta that colocalized with Rh–EGF (arrows). Scale bars: 10 µm. (C,E) Quantification of GFP puncta. The number of GFP puncta was counted per cell. A typical example of an experiment conducted three times is shown. The number of cells examined is indicated. The results are shown as mean±s.d. ***P<0.001 [Welch's *t*-test (C) or Dunnett's multiple-comparisons test (E)]. (D) Effects of Annexin A1 or LRRK1 depletion on the formation of ER–endosome contact sites. HeLa S3 cells treated with control siRNA, annexin A1 (ANX A1) siRNA or LRRK1 siRNA were co-transfected with ERj1(1–200)–V5–GFP_1-10_ and EGFR–GFP_11_. After 16 h of serum starvation, the cells were stimulated with Rh–EGF (50 ng/ml) for 30 min, fixed and immunostained with anti-V5 antibodies (cyan). Images were captured by confocal microscopy and are representative of three experimental repeats. The arrows indicate GFP puncta that colocalized with Rh–EGF. Scale bars: 10 µm.

Encounters between PTP1B, which localizes to the ER membrane, and phosphorylated EGFR, which travels along endosomes, are largely restricted to the MCSs formed between these organelles (Eden et al., 2010; Haj et al., 2002). Thus, MCSs serve to dephosphorylate EGFR and facilitate its sorting into ILVs (Eden et al., 2010; Wong et al., 2018). We hypothesized that the inhibition of LRRK1 signaling disrupts the formation of MCSs between EGFR-containing endosomes and the ER, thereby preventing PTP1B-mediated sorting of EGFR into ILVs. To address this possibility, we examined the effect of LRRK1 depletion on contact formation between the ER and endosomes. ER–endosome contact sites are usually less than 30 nm wide and can only be observed by electron microscopy (Phillips and Voeltz, 2016). We attempted to visualize the contact sites using the split-GFP system, in which GFP is split into two non-fluorescent fragments, the first ten β-strands of GFP (GFP_1-10_) and the last (11th) β-strand (GFP_11_), and spontaneously assemble to form the complete β-barrel structure of fluorescent GFP (Fig. 4A) (Cabantous et al., 2005; Magliery et al., 2005). By targeting each moiety on one of the juxtaposed membranes, GFP fluorescence is restored when the two portions are sufficiently close to one another. We expressed a fusion protein [ERj1(1–200)–V5–GFP_1-10_], in which a V5 tag and a split-GFP fragment were fused to the N-terminal 200 residues of the ER protein ERj1 (Kakimoto et al., 2018), and a fusion protein (EGFR–GFP_11_), in which a split-GFP fragment was fused to the C-terminus of EGFR (Fig. 4A). When co-expressed in HeLa S3 cells, we observed only a few GFP puncta in the absence of EGF stimulation (Fig. 4B,C). Immunofluorescence using anti-V5 antibodies revealed that ERj1(1– 200)–V5–GFP_1-10_ was uniformly distributed on the ER (Fig. 4B). Treating these cells with Rh–EGF for 30 min significantly increased the number of GFP puncta, most of which overlapped with Rh–EGF (Fig. 4B,C). When expressed alone, neither of these constructs generated any detectable fluorescence, despite EGF stimulation (Fig. S4). These results indicate that the split-GFP fragments were properly expressed, targeted and self-assembled. Thus, the split-GFP system is useful for detecting MCSs between the ER and EGFR-containing endosomes.

We next verified whether the split-GFP responds to genetic modulation of the ER–endosome interaction. We depleted annexin A1, a protein identified as a tether between the ER and EGFR-containing endosomes (Eden et al., 2016), to monitor the behavior of the split-GFP. Depletion of annexin A1 (siANX A1) by siRNA in HeLa S3 cells (Fig. S1B) reduced the number of split-GFP foci in the presence of EGF (Fig. 4D,E). Thus, the split-GFP reflects the formation of MCSs between the ER and the endosomes. To determine the contribution of LRRK1 to the formation of ER–endosome contact sites, we compared the number of GFP signals per cell between the control and LRRK1 siRNA-treated cells. We observed that, in contrast to what was seen with annexin A1 depletion, LRRK1 depletion did not decrease the number of EGF-induced GFP signals (Fig. 4D,E). These results suggest that LRRK1 is not required for the formation of MCSs between the ER and EGFR-containing endosomes.

### Dephosphorylation of EGFR Y-1068 at the ER–endosome contact site

**Fig. 5.**
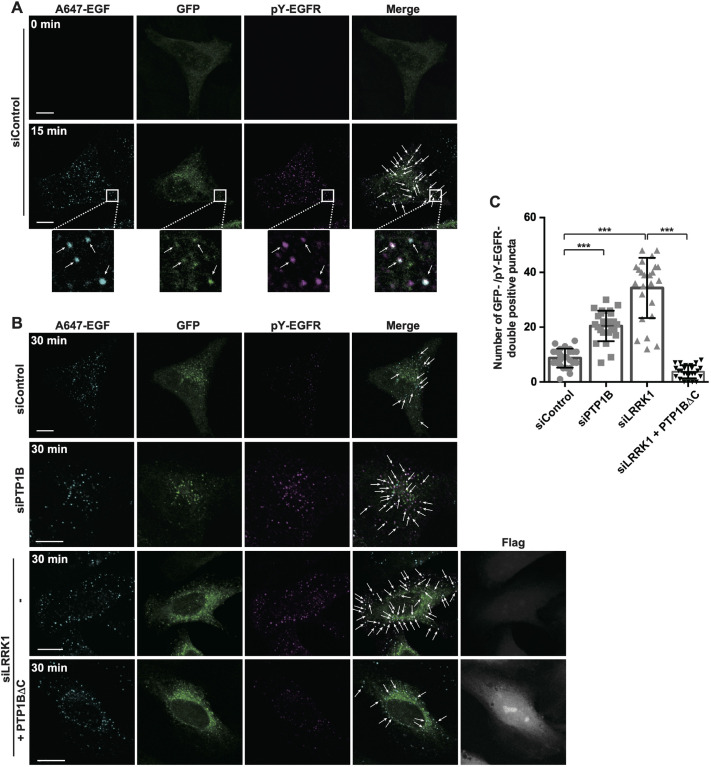
**Dephosphorylation of EGFR Y1068 at the ER–endosome contact site.** (A) Phosphorylation state of EGFR Y1068 at the ER–endosome contact site. HeLa S3 cells treated with control siRNA were co-transfected with ERj1(1–200)–V5–GFP_1-10_ and EGFR–GFP_11_. After 16 h of serum starvation, the cells were stimulated with A647–EGF (50 ng/ml) for 3 min at 37°C, followed by washing to remove labeled EGF from the medium. The cells were fixed at 15 min and immunostained with anti-pY1068-EGFR antibodies (magenta). Images were captured by confocal microscopy and are representative of three experimental repeats. The boxed regions are magnified to show GFP puncta that colocalized with pY1068-EGFR (arrows). Scale bars: 10 µm. (B) Dephosphorylation of EGFR pY1068 at the ER–endosome contact site. HeLa S3 cells treated with control siRNA, PTP1B-specific siRNA or LRRK1-specific siRNA were co-transfected with ERj1(1–200)–V5–GFP_1-10_, EGFR–GFP_11_ and PTP1BΔC. After 16 h of serum starvation, the cells were stimulated with A647–EGF (50 ng/ml) for 3 min at 37°C, followed by washing to remove labeled EGF from the medium. The cells were fixed at 30 min and immunostained with anti-pY1068-EGFR antibodies (magenta) and anti-Flag antibodies (gray). Images were captured by confocal microscopy. The arrows indicate GFP puncta colocalized with pY1068-EGFR. Scale bars: 10 µm. (C) Quantification of GFP puncta colocalized with pY1068-EGFR from cells treated as in B. Data represent the mean±s.d. number of GFP and pY1068-EGFR double-positive puncta per cell from three independent experiments with 25 cells scored per condition. ***P<0.001; n.s., not significant (Dunnett's multiple-comparison test).

How is LRRK1 involved in PTP1B-mediated dephosphorylation of EGFR? First, we confirmed that PTP1B dephosphorylates EGFR pY1068 at the ER–endosome contact site using the split-GFP system. HeLa S3 cells expressing ERj1(1–200)–V5–GFP_1-10_ and EGFR–GFP_11_ were stimulated with Alexa Fluor 647-conjugated EGF (A647-EGF) and immunostained with the pY1068-EGFR antibody. We observed that after EGF stimulation, pY1068-EGFR signals transiently increased on the split-GFP foci overlapping with A647–EGF (Fig. 5A), suggesting that pY1068-EGFR localizes to the ER–endosome contact site in response to EGF stimulation. This phosphorylation peaked 15 min after EGF treatment and disappeared at 30 min. When PTP1B was depleted with siRNA, the number of split-GFP foci in the presence of EGF was not affected (Fig. S5A,B) and pY1068-EGFR signals remained present on the GFP foci, even 30 min following A647–EGF stimulation (Fig. 5B,C). Similar to what was seen with PTP1B siRNA, we found that LRRK1 knockdown sustained accumulation of pY1068-EGFR signal at the ER–endosome contact site (Fig. 5B,C). Furthermore, the overexpression of PTP1BΔC restored the defect in EGFR dephosphorylation caused by LRRK1 depletion (Fig. 5B,C). These results suggest that LRRK1 is required for EGFR pY1068 dephosphorylation by PTP1B at the ER–endosome contact site.

### LRRK1 promotes the EGFR–PTP1B interaction in response to EGF stimulation

**Fig. 6.**
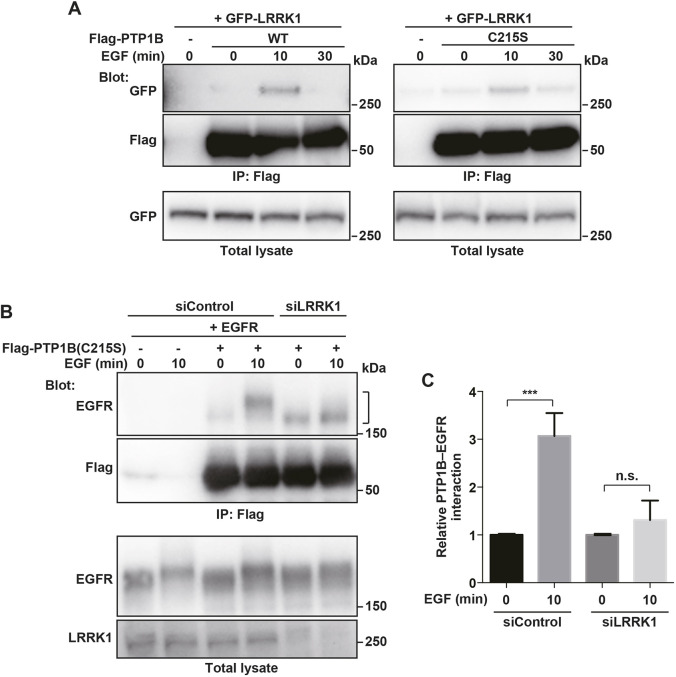
**LRRK1 promotes the EGFR–PTP1B interaction in response to EGF stimulation.** (A) LRRK1 interacts with PTP1B. HEK293 cells were co-transfected with GFP–LRRK1 and Flag–PTP1B or Flag–PTP1B(C215S). After 16 h of serum starvation, cells were stimulated with EGF (50 ng/ml) for the indicated times. Complex formation was detected by immunoprecipitation (IP) with anti-Flag antibodies, followed by immunoblotting (Blot) with anti-GFP antibodies. Total cell lysates (1%) were immunoblotted with the indicated antibodies. Blot is representative of three experimental repeats. (B) Effect of LRRK1 depletion on EGF-induced PTP1B–EGFR interaction. HEK293 cells treated with control or LRRK1 siRNA were co-transfected with EGFR and Flag-PTP1B(C215S). After 16 h of serum starvation, the cells were stimulated with EGF (50 ng/ml) for 0 or 10 min. Complex formation was detected by immunoprecipitation (IP) with anti-Flag antibodies, followed by immunoblotting (Blot) with anti-EGFR antibodies. Total cell lysates (1%) were immunoblotted with the indicated antibodies. (C) Relative levels of EGF-induced PTP1B–EGFR interaction. The amount of EGFR immunoprecipitated with Flag-PTP1B(C215S) was normalized against that of total EGFR. The graph shows the EGFR–PTP1B(C215S) interaction ratio 10 min after EGF stimulation, with 0 min as 1. Data are mean±s.d., combined from three independent experiments. ***P<0.001; n.s., not significant (Welch's *t*-test).

Next, we assessed the role of LRRK1 in the PTP1B-mediated dephosphorylation of EGFR. LRRK1 is likely to act as a scaffold protein for complex formation between EGFR and PTP1B. Given that LRRK1 associates with EGFR via Grb2 (Hanafusa et al., 2011), we examined whether LRRK1 also interacts with PTP1B. HEK293 cells were co-transfected with GFP–LRRK1 and Flag-tagged PTP1B. Co-immunoprecipitation experiments revealed that LRRK1 was transiently associated with PTP1B in a manner dependent on EGF stimulation (Fig. 6A).

We determined the effect of LRRK1 on the EGFR–PTP1B interaction. The association of PTP1B with EGFR represents an enzyme–substrate interaction, and tyrosine phosphorylation of EGFR is required for this interaction (Flint et al., 1997). The PTP1B–EGFR interaction is transient and undetectable by a co-immunoprecipitation assay. In addition, a catalytically inactive PTP1B(C215S) mutant is stable enough to detect interaction with tyrosine-phosphorylated substrates (Flint et al., 1997). We co-expressed EGFR and Flag–PTP1B(C215S) in HEK293 cells and treated the cells with EGF. As expected, Flag–PTP1B(C215S) associated with EGFR, which was dependent upon EGF stimulation (Fig. 6B,C). In contrast, depletion of LRRK1 with siRNA reduced the interaction following EGF stimulation (Fig. 6B,C). Furthermore, LRRK1 bound to PTP1B(C215S) in an EGF stimulation-dependent manner (Fig. 6A). These results suggest that LRRK1 acts as a scaffold protein that promotes the interaction between EGFR and PTP1B in response to EGF stimulation.

### PTP1B dephosphorylates LRRK1 pY944 at the ER–endosome contact site

**Fig. 7.**
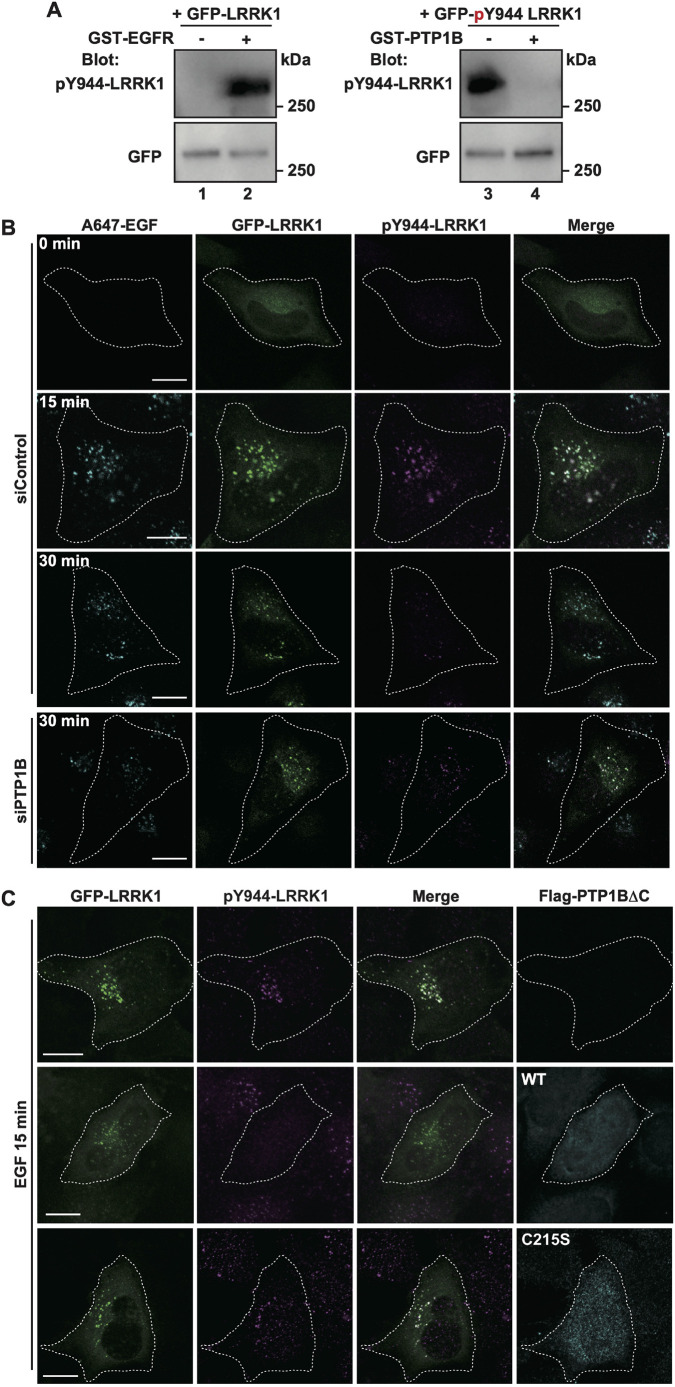
**PTP1B dephosphorylates LRRK1 pY944.** (A) PTP1B dephosphorylates LRRK1 pY944 *in vitro*. Cos7 cells were transfected with GFP–LRRK1 and cell lysates were immunoprecipitated with anti-GFP antibodies. The immunopurified LRRK1 protein was subjected to an *in vitro* kinase assay using recombinant human EGFR protein. Phosphorylated LRRK1 at Y944 was subjected to an *in vitro* phosphatase assay using recombinant human PTP1B protein. Phosphorylated LRRK1 was detected by immunoblotting (Blot) with anti-pY944-LRRK1 antibodies. Equal protein input was confirmed by immunoblotting (Blot) with anti-GFP antibodies. (B,C) PTP1B dephosphorylates LRRK1 pY944 *in vivo*. HeLa S3 cells treated with control siRNA or PTP1B-specific siRNA were transfected with GFP–LRRK1 (B), GFP–LRRK1 plus Flag–PTP1BΔC(WT) (C), and GFP–LRRK1 plus Flag–PTP1BΔC(C215S) (C). After 16 h of serum starvation, the cells were stimulated with A647–EGF (50 ng/ml) (B) or EGF (50 ng/ml) (C) for 3 min at 37°C, followed by washing to remove EGF from the medium. The cells were fixed at 15 or 30 min and immunostained with anti-pY944-LRRK1 antibodies (magenta). Images were captured by confocal microscopy. White dotted lines indicate GFP–LRRK1-expressing cells. Scale bars: 10 µm. Blot and images are representative of three experimental repeats.

We recently demonstrated that EGFR inactivates LRRK1 kinase activity by phosphorylating Y944 (Ishikawa et al., 2012). The non-phosphorylatable LRRK1(Y944F) mutant exhibits hyper-active kinase activity, which results in increased motility and accumulation of EGFR-containing endosome in the perinuclear region (Ishikawa et al., 2012). These findings suggest that LRRK1 kinase activity must be inactive until the EGFR sorting is complete, after which the kinase activity must be activated to transport EGFR-containing endosomes to the perinuclear region. Given that LRRK1 promotes EGFR dephosphorylation by PTP1B at the ER–endosome contact site, it is possible that PTP1B simultaneously dephosphorylates LRRK1 pY944 to activate its kinase activity. To determine whether LRRK1 pY944 is a substrate for PTP1B, Cos7 cells were transfected with GFP–LRRK1, and cell extracts were immunoprecipitated with anti-GFP antibodies. Immunopurified GFP–LRRK1 was incubated with recombinant EGFR protein, followed by immunoblotting with anti-pY944-LRRK1 antibodies. Consistent with our previous results (Ishikawa et al., 2012), EGFR phosphorylated LRRK1 on Y944 *in vitro* (Fig. 7A, lanes 1,2). We evaluated tyrosine dephosphorylation of GFP–LRRK1 pY944 *in vitro* in the presence or absence of active recombinant PTP1B protein. We found that LRRK1 pY944 was dephosphorylated only in the presence of PTP1B (Fig. 7A, lanes 3,4), indicating that LRRK1 is a substrate for PTP1B.

Finally, we examined whether PTP1B dephosphorylates the pY944 residue of LRRK1 at the ER–endosome contact site. HeLa S3 cells were transfected with GFP–LRRK1 and stimulated with A647–EGF. As observed previously (Hanafusa et al., 2011), GFP–LRRK1 was distributed in a punctate pattern and colocalized with A647–EGF (Fig. 7B). This indicates that endocytosed GFP–LRRK1 localizes to the endosomes. Immunostaining with anti-pY944-LRRK1 antibodies revealed that pY944-LRRK1 signals were detected on the GFP–LRRK1 puncta 15 min after A647–EGF stimulation (Fig. 7B). However, no pY944-LRRK1 signal was observed in HeLa S3 cells expressing the non-phosphorylatable GFP-LRRK1(Y944F) mutant (Fig. S6). Thus, the anti-pY944-LRRK1 antibody specifically recognizes Y944-phosphorylated LRRK1. The Y944 phosphorylation of LRRK1 disappeared 30 min after EGF stimulation (Fig. 7B), indicating that the increase in pY944-LRRK1 signal was transient. When PTP1B was depleted with siRNA, pY944-LRRK1 signals remained on the GFP–LRRK1 puncta, even 30 min after stimulation (Fig. 7B). Furthermore, co-expression of PTP1BΔC with GFP–LRRK1 significantly reduced the pY944 signal on GFP–LRRK1 induced by EGF stimulation (Fig. 7C). By contrast, in cells overexpressing the catalytically inactive PTP1BΔC(C215S) mutant, the level of pY944 on GFP–LRRK1 remained elevated (Fig. 7C), indicating that pY944 dephosphorylation depends on the tyrosine phosphatase activity of PTP1BΔC. Using the split-GFP system, we investigated whether the pY944-LRRK1 signal was present at the ER–endosome contact site. HeLa S3 cells carrying ERj1(1–200)–V5–GFP_1-10_ and EGFR–GFP_11_ were stimulated with A647–EGF, followed by immunostaining with the pY944-LRRK1 antibody. We found that several endogenous pY944-LRRK1 signals were localized on the split-GFP foci overlapping with A647–EGF (Fig. S7). Taken together, these results suggest that PTP1B dephosphorylates pY944 on LRRK1 at the ER–endosome contact site.

## DISCUSSION

To maintain cellular homeostasis after receptor activation, a mechanism is required to attenuate the signal when the stimulus is no longer present. In the case of receptor tyrosine kinases, it has become clear that they are regulated at multiple levels, including not only dephosphorylation but also receptor endocytosis and degradation (Citri and Yarden, 2006). The role of the endocytic pathway in the regulation of receptor signaling is now well established, and the deregulation of receptor endocytosis might lead to altered signaling and cellular transformation (Tomas et al., 2014). The cellular response to EGF is tightly regulated through the control of receptor trafficking and activation state (Bakker et al., 2017), with EGFR tyrosine phosphorylation being controlled by the dynamic interplay between kinases and phosphatases (Schlessinger, 2000). Our previous studies revealed a role for LRRK1 kinase, which modulates EGFR signaling by regulating the components of the endocytic machinery. We have shown that LRRK1 knockdown inhibits EGFR sorting into endosomal ILVs, resulting in the retention of clustered EGFR on the limiting membrane of endosomes (Hanafusa et al., 2011). In the present study, we demonstrated that LRRK1 provides directionality to EGFR sorting into ILVs by inducing EGFR tyrosine dephosphorylation. Mechanistically, our findings suggest that LRRK1 functions as a scaffold protein for the EGFR–PTP1B interaction by binding to EGFR and PTP1B, which results in enhanced dephosphorylation of EGFR by PTP1B at the ER–endosome contact site and subsequent sorting of EGFR into the endosomal lumen. Furthermore, LRRK1 depletion maintains the receptor accumulated on the endosomes in a phosphorylated active form, leading to sustained MAPK pathway activation.

Eden et al. (2010) identified a role for PTP1B, which localizes to the ER, in EGFR sequestration into ILVs. PTP1B suppresses EGFR signaling, not only by dephosphorylating EGFR but also by promoting its sorting into ILVs (Haj et al., 2003; Eden et al., 2010), a process that sequesters the catalytic domain of the receptor from its cytoplasmic substrates before degradation in the lysosomes. Thus, activated EGFR molecules undergo internalization into endosomes, where they continue to signal in a phosphorylated state until dephosphorylation by PTP1B on the ER. Here, we found that LRRK1 depletion reduces EGFR dephosphorylation by PTP1B. Importantly, the expression of the cytosolic domain of PTP1B (PTP1BΔC) in LRRK1 siRNA-treated cells rescues delayed EGFR sorting into ILVs. Furthermore, we demonstrated that EGF stimulation induces the binding of EGFR to PTP1B in an LRRK1-dependent manner. These results are consistent with a model in which LRRK1-mediated complex formation between EGFR and PTP1B contributes to the attenuation of EGFR-mediated signaling.

MCSs are usually microdomains in close membrane apposition associated with a diverse range of functionally distinct contact sites. This provides an important means of non-vesicular communication between organelles (Phillips and Voeltz, 2016). At ER–endosome contact sites, ER-localized PTP1B interacts with and dephosphorylates activated EGFR to promote receptor sorting into ILVs (Eden et al., 2010; Wong et al., 2018). These MCSs might provide a platform for the tight control of the EGFR phosphorylation state. Contact sites are maintained by tethering organelles to draw the membranes into proximity. In the case of MCSs between ER and EGFR-containing endosomes, annexin A1 acts as an important tether (Eden et al., 2016). Here, we used the split-GFP system to monitor MCSs by confocal fluorescence microscopy. We constructed split-GFP probes using the ER-localized protein ERj1 and EGFR itself. These split-GFP probes specifically detect MCSs between the ER and EGFR-containing endosomes. The split-GFP probes formed GFP puncta, which was dependent upon EGF stimulation, and the knockdown of annexin A1 significantly reduced the number of GFP puncta. In contrast, LRRK1 depletion had no inhibitory effect on the number of GFP puncta, suggesting that LRRK1 is dispensable for the formation of MCS between ER and EGFR-containing endosomes. Because LRRK1 is required for the EGF-mediated EGFR–PTP1B interaction, it is likely that LRRK1 functions as a scaffold protein during EGFR dephosphorylation by PTP1B after the formation of ER–endosome contact sites. Consistent with this, LRRK1 depletion inhibits EGFR pY1068 dephosphorylation by PTP1B at the ER–endosome contact site.

Our findings indicate that LRRK1 has two functions in regulating EGFR trafficking events (Hanafusa et al., 2011, 2019; Ishikawa et al., 2012; Kedashiro et al., 2015). First, LRRK1 regulates the trafficking of EGFR-containing endosomes, which depends on its kinase activity. Second, LRRK1 regulates the sorting of EGFR into ILVs in a kinase activity-independent manner. The transport of EGFR-containing endosomes is coordinated with the early-to-late maturation of endosomes and their eventual fusion with lysosomes (Ishikawa et al., 2012). The expression of an activated form of LRRK1, LRRK1(Y944F), results in the transport of EGFR-containing endosomes to the nuclear region in an immature form. Thus, LRRK1(Y944F) increases EGFR trafficking but is less effective compared with wild-type LRRK1 in promoting EGFR sorting into ILVs. In other words, EGFR activated by EGF stimulation inactivates the LRRK1 kinase activity by phosphorylating Y944, and the inactive form of LRRK1 mediates the sorting of EGFR into ILVs. If the LRRK1 kinase activity is constitutively activated, the EGFR-containing endosomes are transported before EGFR is sorted into ILVs. After EGFR is sorted into ILVs, LRRK1 must be activated by pY944 dephosphorylation at this stage because the kinase activity of LRRK1 is required for the trafficking of EGFR-containing endosomes. The binding of PTP1B to LRRK1 likely causes PTP1B to activate LRRK1 by dephosphorylating pY944. Indeed, we demonstrated that PTP1B dephosphorylates LRRK1 pY944 on the ER–endosome contact site. Thus, the MCS between the endosome and ER functions as a hub for LRRK1-dependent signaling.

## MATERIALS AND METHODS

### Cell cultures, antibodies and reagents

HeLa S3, HEK293, and Cos7 cells were cultured in DMEM containing 10% FBS. These cell lines were obtained from the American Type Culture Collection or the Japanese Collection of Research Bioresources and regularly tested for *Mycoplasma* contamination. The antibodies and suppliers were as follows: anti-pY1068-EGFR [cat. no ab32430, Abcam or cat. no 2234, Cell Signaling Technology (CST)]; anti-EGFR (cat. no MI-12-1, MBL); anti-phospho-ERK (cat. no 9106, CST); anti-ERK (cat. no sc-94, Santa Cruz Biotechnology); anti-GFP (598, MBL); anti-Flag (M2, cat. no F1804, Sigma or FLA-1, cat. no M-185-3L, MBL); anti-V5 (cat. no 46-0705, Invitrogen); anti-PTP1B (cat. no ab252928, Abcam); and anti-annexin A1 (cat. no ab135256, Abcam). Affinity-purified rabbit antibodies against pY944-LRRK1 were produced according to a previously described method (Ishikawa et al., 2012). Rh–EGF and EGF were purchased from Sigma. A647–EGF was purchased from Thermo Fisher Scientific. Full uncropped immunoblot images are shown in Fig. S8.

### Plasmids, mutations and RNA interference

Human LRRK1 was cloned from a cDNA library by PCR (the clone lacks 27 amino acids at the N-terminus compared with NM_024652). GFP–LRRK1 and GFP–Rab5(Q79L) were generated as described previously (Hanafusa et al., 2011). PTP1B(C215S), PTP1BΔC and PTP1BΔC(C215S) were created using the QIAquick mutagenesis kit according to the manufacturer's protocol (Qiagen) and subcloned into the pCMV-Flag vector (635688, Takara). ERj1(1–200)–V5–GFP_1-10_ was generated as previously described (Kakimoto et al., 2018). EGFR was subcloned into the GFP_11_ vector. siRNA for human LRRK1 [target sequence, 5′-GCAGGAACAGGAAAGTCACCATTTA(tt)-3′], human LRRK1 a#1 [target sequence, 5′-GGAATCACTCACTGACTAC(tt)-3′], human PTP1B [target sequence, 5′-GGAGAAAGGTTCGTTAAAA(tt)-3′], and human annexin A1 [target sequence, 5′-AATCCATCCTCGGATGTCGCT(tt)-3′] were purchased from Ambion and Life Technologies or JBioS. Control 2 siRNA (Silencer Select; Life Technologies) was used as a negative control. Annealed siRNAs were transfected using RNAiMAX (Invitrogen) and the transfected cells were analyzed 72 h after transfection.

### Immunoprecipitation

For immunoprecipitation, the cells were lysed in RIPA buffer [50 mM Tris-HCl (pH 7.4), 0.15 M NaCl, 0.25% deoxycholic acid, 1% NP-40, 1 mM EDTA, 1 mM dithiothreitol, phosphatase inhibitor cocktail 2 (Sigma), and protease inhibitor cocktail (Sigma)], followed by centrifugation at 15,000* **g*** for 12 min. The supernatant was added to 50 µl (1.5 mg) of Dynabeads Protein G (Invitrogen) with the anti-Flag antibodies (M2, 10 µg) and rotated for 2 h at 4°C. The beads were then washed three times with ice-cold phosphate-buffered saline and subjected to immunoblotting.

### Immunofluorescence and image analysis

For immunofluorescence staining, the cells were grown on coverslips, treated as indicated, fixed in 4% paraformaldehyde for 15 min at 37°C, permeabilized in 0.5% Triton X-100 for 5 min, and incubated with primary and secondary antibodies. Primary antibodies were as follows: mouse anti-Flag at 1:500, anti-V5 at 1:1000, anti-EGFR at 1:250, rabbit anti-pY1068-EGFR at 1:200, and anti-pY944-LRRK1 at 1:2500. The secondary antibodies used were as follows: Alexa Fluor 405-, 488-, 555- or 647-conjugated goat anti-mouse-IgG or anti-rabbit-IgG antibodies (Invitrogen). Confocal microscopy was undertaken using an Olympus FV3000 microscope. To quantify the number of GFP puncta, the fluorescence intensity of these molecules was measured and counted using the ImageJ plugin (National Institutes of Health, Bethesda, MD, USA). For each series of experiments, the microscope settings were optimized for the brightest, unsaturated images and remained unaltered during analysis.

### Dephosphorylation of LRRK1 pY944 by PTP1B

Recombinant GST–EGFR and GST–PTP1B were purchased from SignalChem. The GFP–LRRK1 proteins were expressed in COS7 cells and immunopurified with anti-GFP antibodies (Ishikawa et al., 2012). A non-radioactive *in vitro* kinase assay using GST–EGFR was performed in a final volume of 20 µl solution consisting of 50 mM HEPES pH 7.4, 5 mM MgCl_2_, 0.5 mM DTT and 200 µM ATP. Samples were incubated for 5 min at 30°C and washed three times with ice-cold phosphate-buffered saline. The washed beads were mixed with or without GST–PTP1B in a phosphatase reaction buffer (25 mM HEPES pH 7.2, 50 mM NaCl, 2.5 mM EDTA, 1 mM DTT and 10 ng/µl BSA), incubated for 20 min at 37°C, and terminated by the addition of Laemmli sample buffer, followed by boiling. The samples were resolved by SDS-PAGE and subjected to immunoblotting.

### Statistical analysis

Statistical analysis was performed using either Dunnett's multiple-comparisons test or Welch's *t*-test. Error bars represent the s.d. Detailed *n* values for each panel in the figures are stated in the corresponding legends. No statistical method was used to pre-determine the sample size. Prism software (GraphPad Software) was used for the analysis of statistical significance.

## Supplementary Material

10.1242/joces.260566_sup1 Supplementary information
