# Supplementary material for: LRRK1 functions as a scaffold for PTP1B-mediated EGFR sorting into ILVs at the ER–endosome contact site
Source: J Cell Sci. 2023 Mar 2;136(6):jcs260566. doi: 10.1242/jcs.260566 (PMC10022742; doi:10.1242/jcs.260566)
Supplement: Supplementary information [file joces-136-260566-s1.pdf]

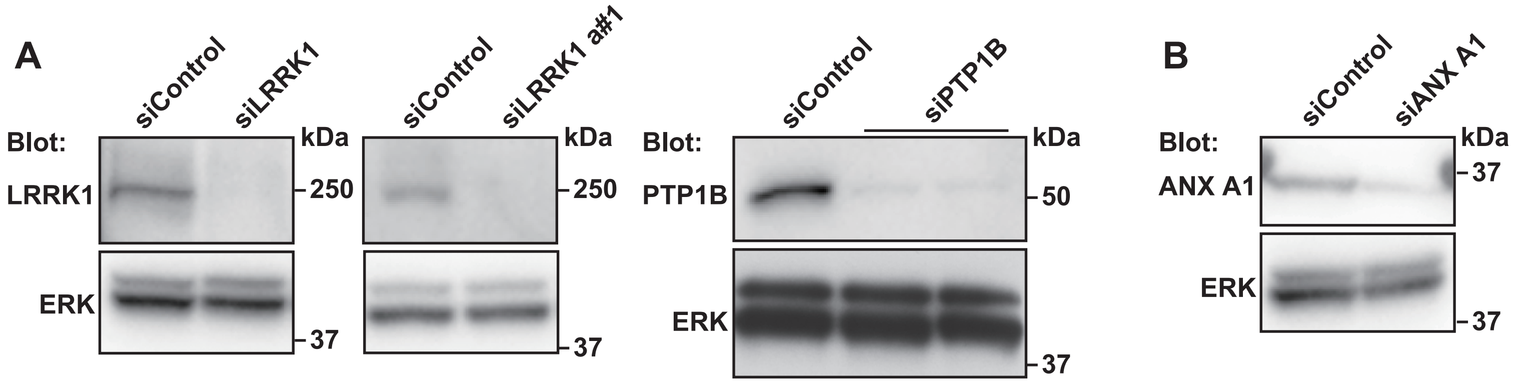

Fig. S1. Effect of siRNA.

(A,B) HeLa S3 cells were treated with control siRNA, LRRK1 siRNA or LRRK1 siRNA a#1 (**A**), PTP1B siRNA (A), or Annexin A1 (ANX A1) siRNA (**B**). Total cell lysates were immunoblotted (Blot) with the indicated antibodies. ERK is served as a loading control.

Journal of Cell Science • Supplementary information

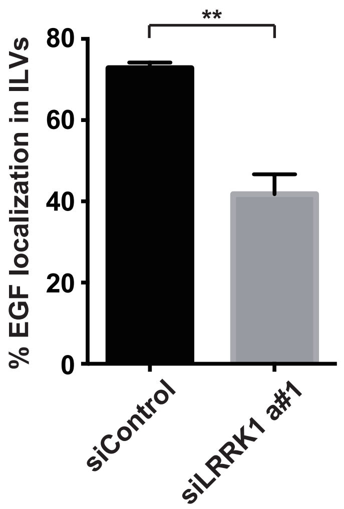

Fig. S2. Effect of LRRK1 siRNA a#1 on EGFR sorting into ILVs.

Quantification of Rh-EGF localization into the endosomal lumen. HeLa S3 cells treated with control siRNA or LRRK1 siRNA a#1 were transfected with GFP-Rab5(Q79L). After 16 h of serum starvation, the cells were stimulated with Rh-EGF (50 ng/ml) for 30 min and fixed. Data are presented as the percentage of Rh-EGF localized into the ILVs of the endosomes out of the total number of endosomes (diameter; >1 µm) and quantified by Welch' s t-test, **P < 0.01. Values reflect the mean SD of three independent experiments with an average of 25 cells (>75 endosomes) scored per experiment.

Journal of Cell Science • Supplementary information

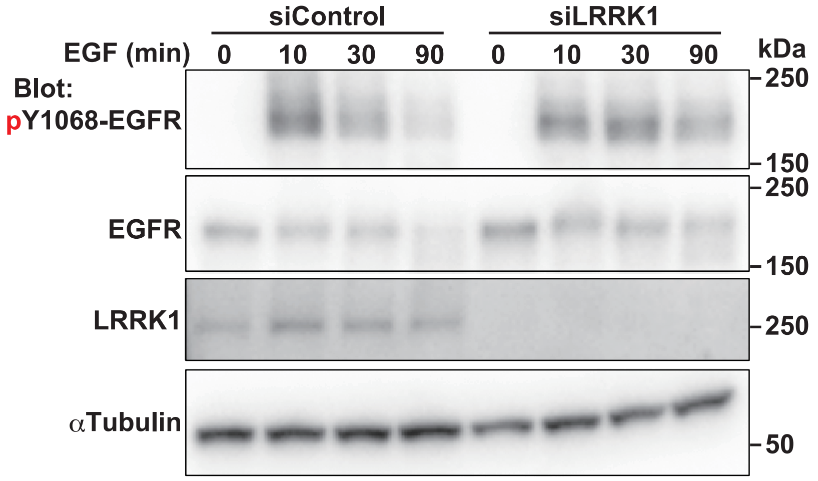

Fig. S3. Effect of LRRK1 depletion on phosphorylation of EGFR Tyr-1068 in response to EGF stimulation.

HeLa S3 cells were treated with control or LRRK1 siRNA. After 16 h of serum starvation, the cells were stimulated with EGF (10 ng/ml) for the indicated times. Cell lysates were prepared and immunoblotted (Blot) with the indicated antibodies. Tubulin serves as a loading control.

Journal of Cell Science • Supplementary information

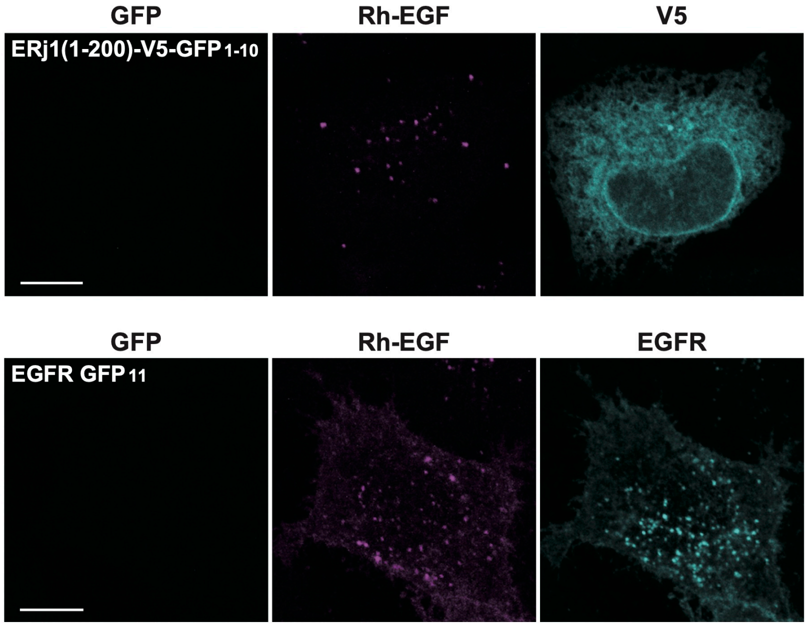

Fig. S4. Formation of ER–endosome contact sites.

HeLa S3 cells were transfected with either ERj1(1–200)-V5-GFP1-10 or EGFR-GFP11. After 16 h of serum starvation, the cells were stimulated with Rh-EGF for 30 min, fixed, and immunostained with anti-V5 (cyan) and anti-EGFR antibodies (cyan) as indicated. Scale bar: 10 µm.

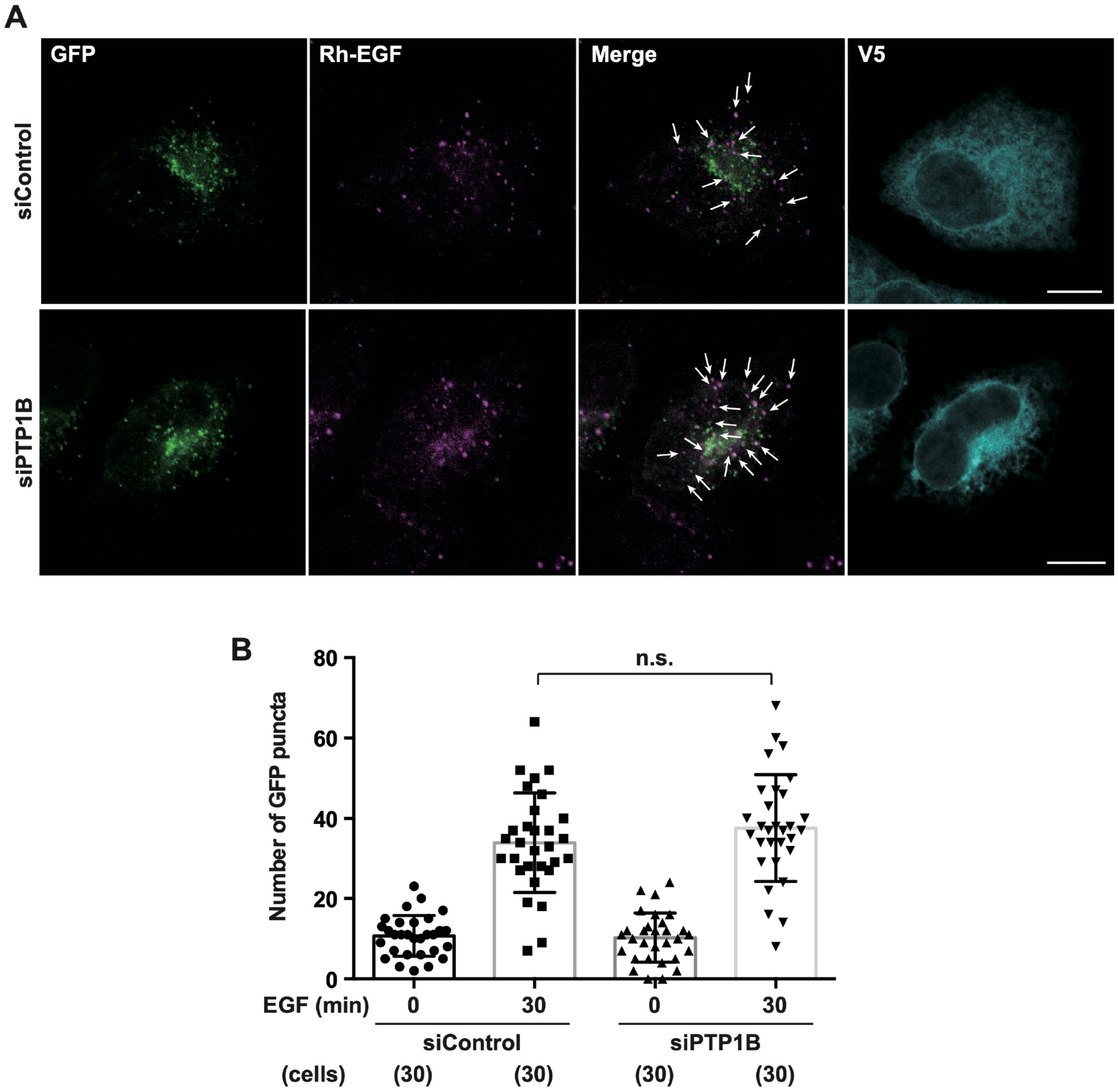

Fig. S5. Effect of PTP1B depletion on EGF-induced ER-endosome contact site formation.

(A) Hela S3 cells treated with control siRNA or PTP1B siRNA were co-transfected with ERj1(1-200)-V5-GFP1-1 O and EGFR-GFP11. After 16 h of serum starvation, the cells were stimulated with Rh-EGF (50 ng/ml) for 30 min, fixed, and immunostained with anti-VS antibodies (cyan). Images were captured by confocal microscopy. The arrows indicate GFP puncta that co-localized with Rh-EGF. Scale bar: 10 μm.

(B) Quantification of GFP puncta. The number of GFP puncta was counted per cell and quantified by Welch' s t-test, n.s., not significant. The number of cells examined is indicated. The error bars represent SD. A typical example of an experiment conducted three times is shown.

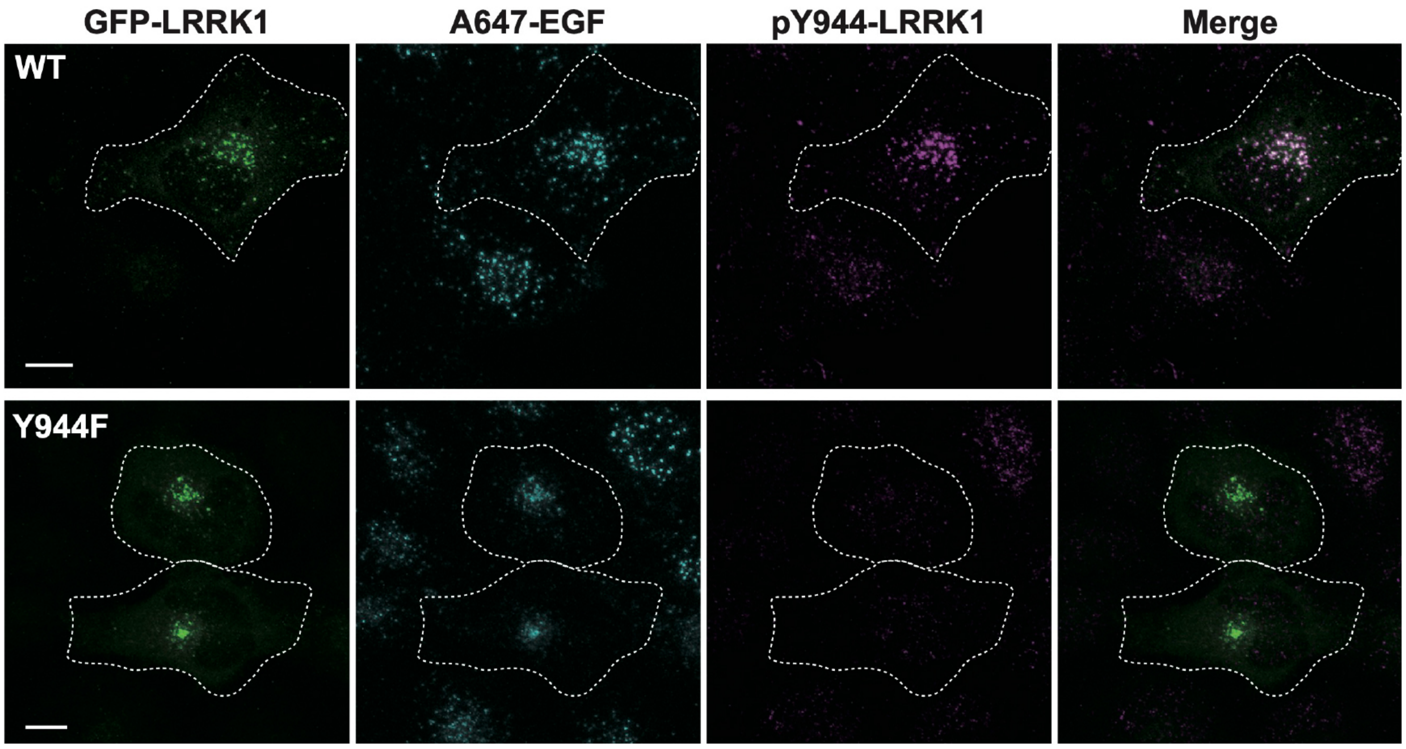

Fig. S6. Specificity of the anti-pV944-LRRK1 antibody.

Hela 83 cells were transfected with GFP-LRRK1 (WT or Y944F). After 16 h of serum starvation, the cells were stimulated with A647-EGF (50 ng/ml) for 20 min, fixed, and immunostained with anti-pY944-LRRK1 antibodies (magenta). Images were captured by confocal microscopy. White dotted lines indicate GFP-LRRK1-expressing cells. Scale bar: 1O µm.

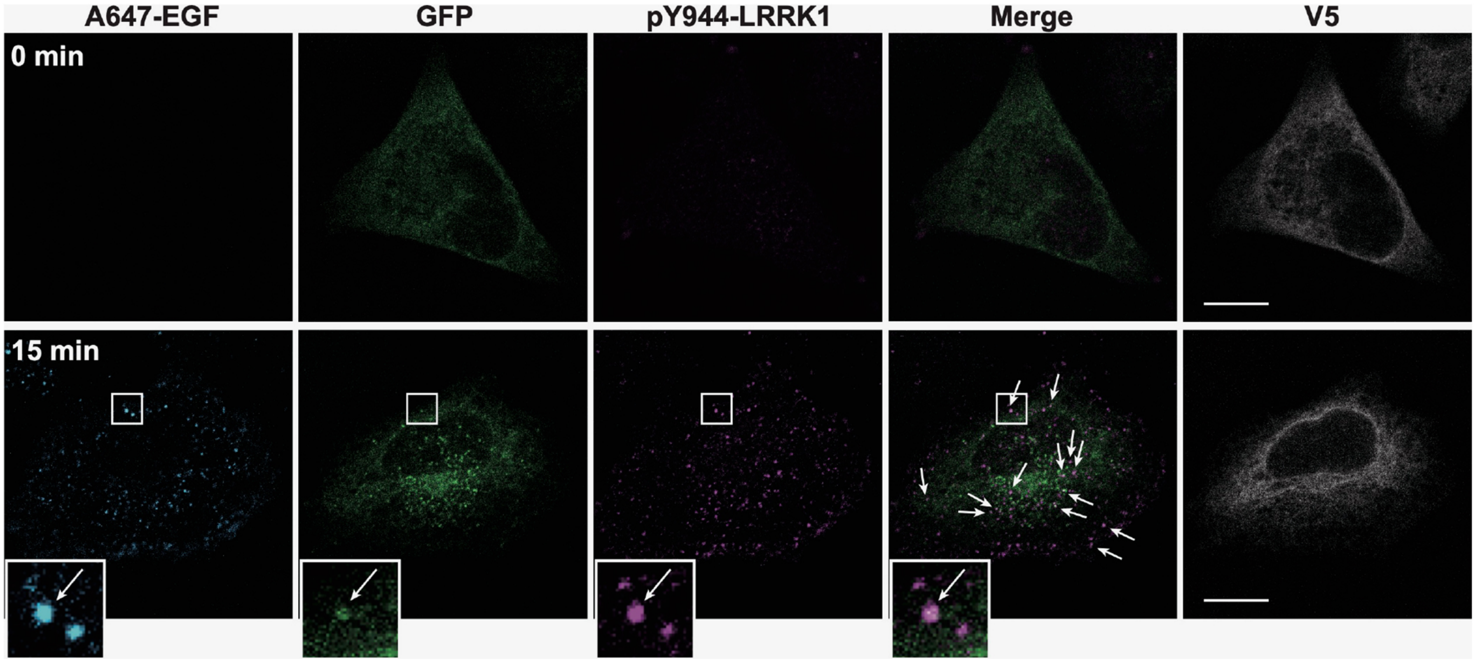

Fig. S7. Localization of pV944-LRRK1 signal at the ER-endosome contact site.

Hela 83 cells were co-transfected with ERj1(1-200)-V5-GFP1-10 and EGFR-GFP11. After 16 h of serum starvation, the cells were stimulated with A647-EGF (50 ng/ml) for 3 min at 37°C, followed by washing to remove labeled EGF from the medium. The cells were fixed at 15 min and immunostained with anti-pY944-LRRK1 antibodies (magenta). Images were captured by confocal microscopy.

The boxed regions are magnified to show GFP puncta that co-localized with pY944-LRRK1 (arrows). Scale bar: 10 µm.

Related to Figure 2: Uncropped scans of western blots.

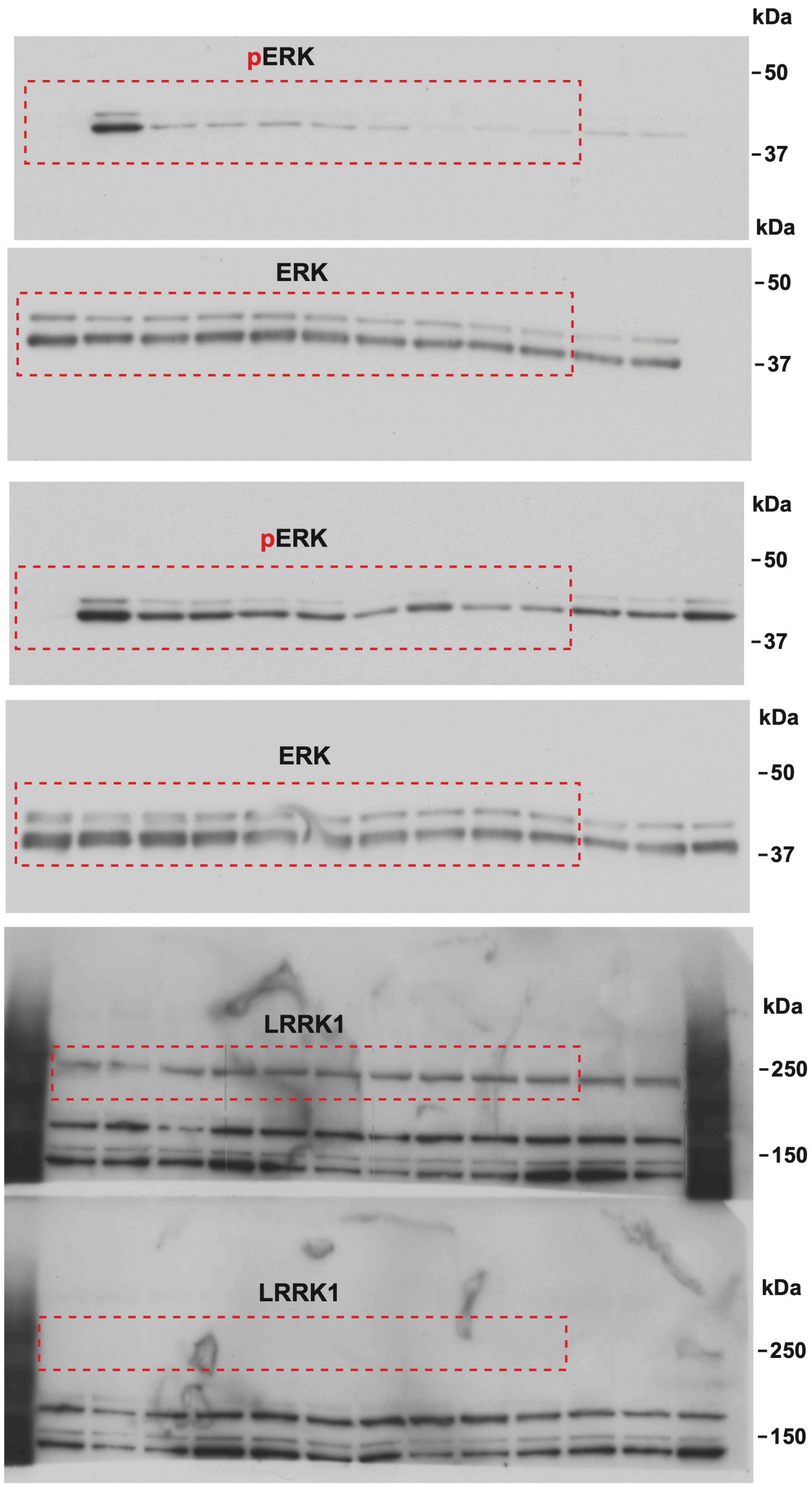

Fig. S8. Blot transparency

Related to Figure 6: Uncropped scans of western blots.

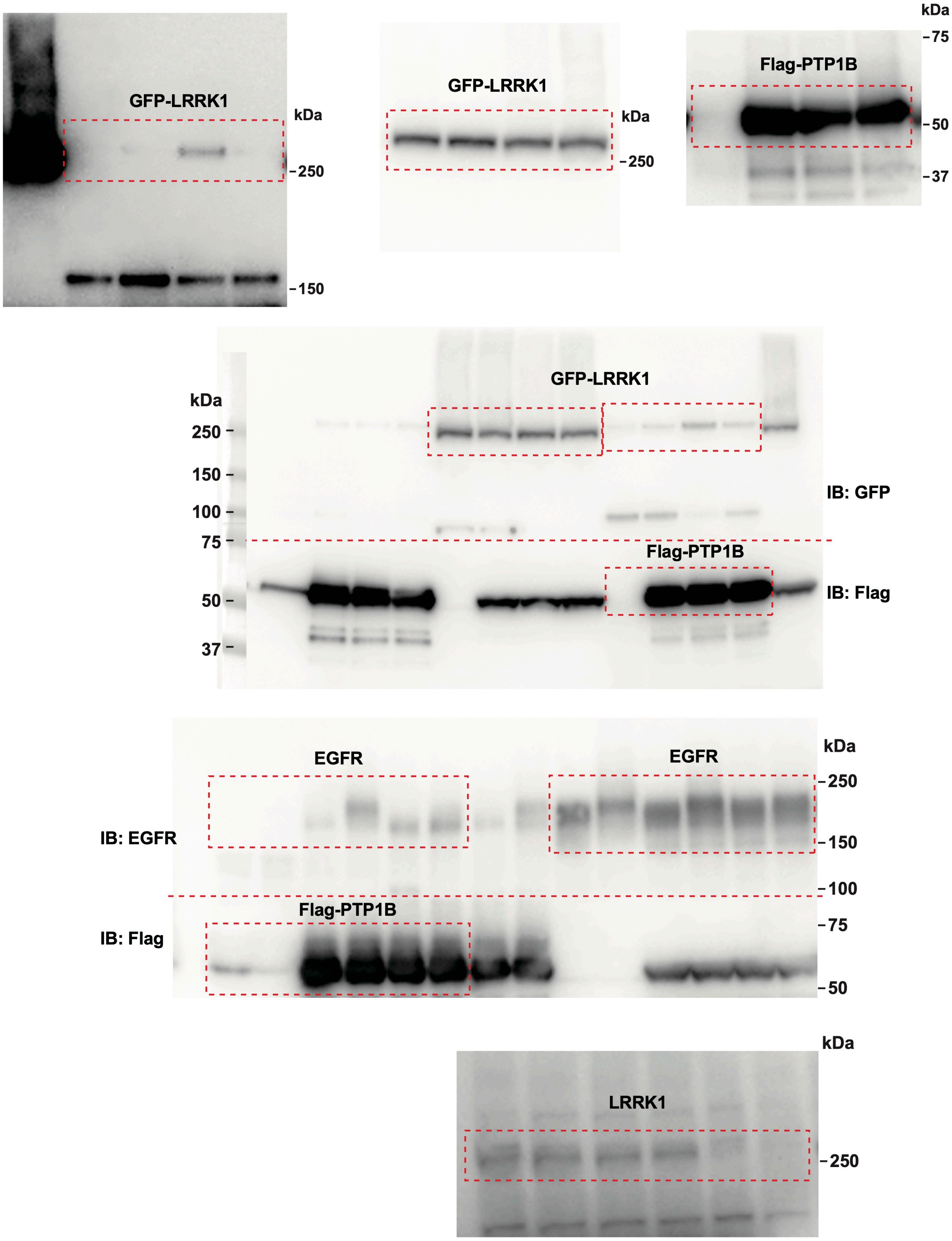

Fig. S8. Blot transparency

Journal of Cell Science • Supplementary information

Related to Figure 7: Uncropped scans of western blots.

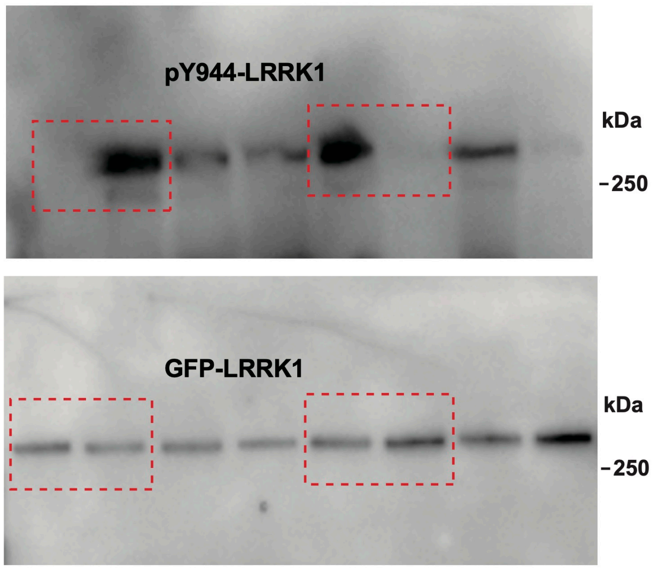

Related to Figure S3: Uncropped scans of western blots.

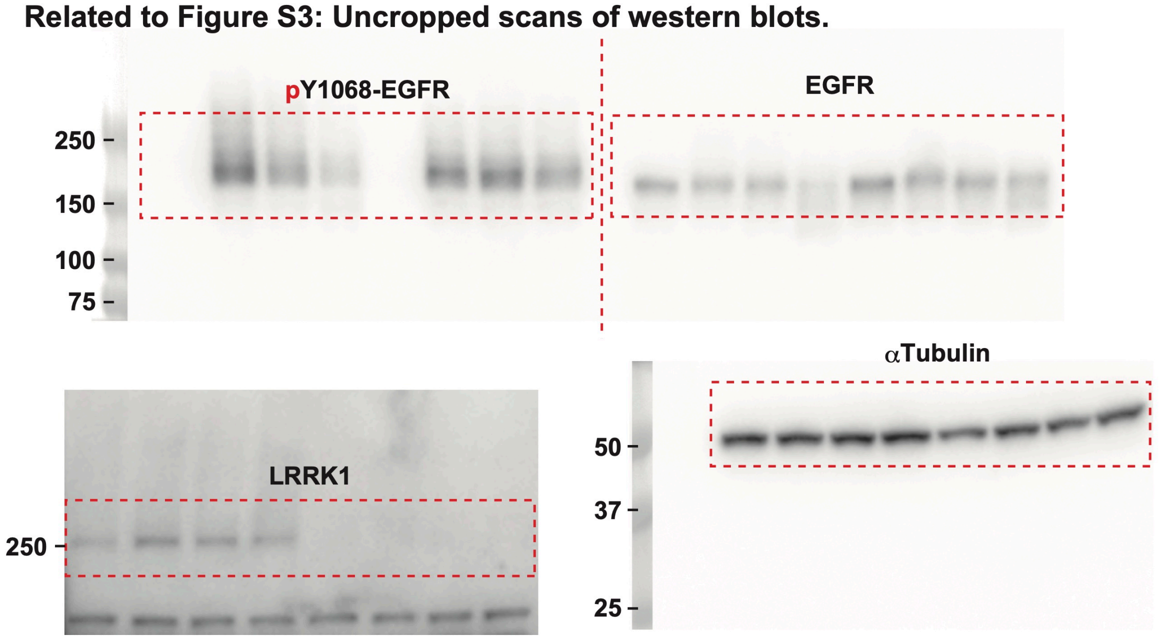

Fig. S8. Blot transparency

Journal of Cell Science • Supplementary information
