# Supplementary material for: LRRK1 functions as a scaffold for PTP1B-mediated EGFR sorting into ILVs at the ER–endosome contact site
Source: J Cell Sci. 2023 Mar 2;136(6):jcs260566. doi: 10.1242/jcs.260566 (PMC10022742; doi:10.1242/jcs.260566)
Supplement: Supplementary file 1 [file joces-136-260566_review_history.pdf]

LRRK1 functions as a scaffold for PTP1B-mediated EGFR sorting into ILVs at the ER—endosome contact site

Hiroshi Hanafusa, Keitaro Fujita, Misa Kamio, Shiori Iida, Yasushi Tamura, Naoki Hisamoto and Kunihiro Matsumoto

DOI: 10.1242/jcs.260566

Editor: Tamotsu Yoshimori

Review timeline

Original submission:	24 August 2022
Editorial decision:	26 September 2022
First revision received:	2 January 2023
Accepted:	30 January 2023

Original submission

First decision letter

MS ID#: JOCES/2022/260566

MS TITLE: LRRK1 functions as a scaffold for PTP1B-mediated EGFR sorting into ILVs at the ER—endosome contact site

AUTHORS: Hiroshi Hanafusa, Keitaro Fujita, Misa Kamio, Shiori Iida, Yasushi Tamura, Naoki Hisamoto, and Kunihiro Matsumoto
ARTICLE TYPE: Research Article

We have now reached a decision on the above manuscript.

To see the reviewers' reports and a copy of this decision letter, please go to: https://submit-jcs.biologists.org and click on the 'Manuscripts with Decisions' queue in the Author Area. (Corresponding author only has access to reviews.)

As you will see, the reviewers raise a number of substantial criticisms that prevent me from accepting the paper at this stage. They suggest, however, that a revised version might prove acceptable, if you can address their concerns. If you think that you can deal satisfactorily with the criticisms on revision, I would be pleased to see a revised manuscript. We would then return it to the reviewers.

Please ensure that you clearly highlight all changes made in the revised manuscript. Please avoid using 'Tracked changes' in Word files as these are lost in PDF conversion.

I should be grateful if you would also provide a point-by-point response detailing how you have dealt with the points raised by the reviewers in the 'Response to Reviewers' box. Please attend to all of the reviewers' comments. If you do not agree with any of their criticisms or suggestions please explain clearly why this is so.

Reviewer 1

Advance summary and potential significance to field

© 2023. Published by The Company of Biologists under the terms of the Creative Commons Attribution License (https://creativecommons.org/licenses/by/4.0/).

In the present study Hanafusa and colleagues demonstrated that LRRK1 is required for EGFR dephosphorylation by ER-localized PTP1B, which occurs at ER-endosome contact sites, followed by sorting of dephosphorylated/inactivated EGFR into the intraluminal vesicles of endosomes. LRRK1 does not participate in EGF-induced formation of the ER–endosome contact sites, but does function as a scaffold that mediates the interaction of PTP1B with phosphorylated EGFR, leading to down-regulation of EGF signaling.

This is a well-written paper and provides a new insight into the function of LRRK1, and thus is a very good candidate for publication in JCS after the following points are adequately answered.

Comments for the author

Major points:

Fig. 6B: There are a couple of concerns about this figure. First, it appears that the amount of phosphorylated EGFR in siLRRK1-treated cells (right lane in the “EGFR, Total lysates” panel) was a bit smaller than that in siControl cells (3rd lane from the right), raising the possibility that no binding of EGFR to Flag-PTP1B(C215S) in siLRRK1-treated cells is due to a smaller amount of phospho-EGFR produced. Regarding this, why was an anti-pY1068-EGFR antibody not used for immunoblotting? Because the anti-EGFR antibody gave fuzzy bands, likely reflecting the formation of phosphorylated forms, use of an anti-pY1068-EGFR antibody may give a clearer result. Use of the latter antibody could also provide direct evidence for sustained activation of EGFR in LRRK1-depleted cells (Fig. 2). Second, does Flag-PTP1B(C215S) bind to LRRK1 with a similar affinity to Flag-PTB1B wild-type (Fig. 6A)? This should be examined because Flag-PTP1B(C215S) was used for binding experiments (Fig. 6B).

Fig. 7B: Does pY944-LRRK1 staining disappear for a longer incubation? It is important to show LRRK1 can be dephosphorylated by endogenous PTP1B.

Fig. 7C: Is pY944-LRRK1 present at ER-endosome contact sites? The localization of pY944-LRRK1 seems to be different from that of pY-EGFR (Fig. 5).

It is better to show that expression of an siRNA-resistant LRRK1 construct can compensate for the phenotype of LRRK1-depleted cells. Alternatively, another siRNA should be tested to exclude the possible off-target effects.

Minor points:

Describe how siRNA-resistant Flag-PTP1B constructs were made.

Fig. 3B-D: How was quadruple staining performed? Because GFP fluorescence overlaps with Alexa-Fluor 488 fluorescence, another fluorescence probe, in addition to Alexa-Fluor 555 and Alexa-Fluor 647, must have been used.

Fig. 4A: “ERj(1-220) ---" should read “ERj1(1-220) ---“.

Fig. 5D: How were PTP1B△C-expressing cells identified? Like Fig. 3B-D, quadruple staining must have bee performed.

<u>Reviewer 2</u>

Advance summary and potential significance to field

In this paper the authors build on their previous observations (confirmed in the current study) that LRRK1 regulates the sorting of EGFR onto intraluminal vesicles (ILVs) of MVBs. They show that LRRK1 regulates the PTP1B-mediated EGFR dephosphorylation that occurs at membrane contact sites between EGFR-containing endosomes and the ER. They further show that LRRK1 is not required for contact site formation using a very elegant split GFP assay that they have validated

© 2023. Published by The Company of Biologists under the terms of the Creative Commons Attribution License (https://creativecommons.org/licenses/by/4.0/).

using a known membrane contact site regulator, annexin 1. They then present evidence that LRRK1 acts as a scaffold to enable EGFR:PTP1B interaction.

One of the most interesting parts of this study is the idea that LRRK1 has 2 roles in EGFR trafficking that are regulated by its phosphorylation. They had previously shown that EGFR phosphorylates LRRK1 on a site that inhibits LRRK1 kinase activity and that LRRK1 kinase activity though not involved in sorting of EGFR onto ILVs is required for movement of EGFR-containing endosomes to the perinuclear region.

Here they show that LRRK1 is a substrate of PTP1B. A potential order of events is presented whereby EGFR phosphorylates and inactivates LRRK1 and LRRK1 acts as a scaffold at membrane contact sites to allow PTP1B and EGFR to interact and drive sorting of EGFR onto ILVs. PTP1B then dephosphorylates LRRK1 allowing the LRRk1 kinase activity to promote movement of EGFR containing endosomes to the perinuclear region. This is a very interesting and novel model and definitely of potential interest to readers of J Cell Science.

Comments for the author

Some very nice approaches have been used in this study. However quantitation of effects on EGFR and LRRK1 phosphorylation is lacking in some places as detailed below, as well as additional points.

Fig. 1A: Endosomes appear larger in the controls than the LRRK1 and PTP1B siRNA-treated cells. Is this the case? If anything I would expect that inhibition of ILV formation would lead to enlarged MVBs. The images should be shown at the same magnification and if the changes in endosome size are consistent then they should be commented upon.

Figure 2 shows the effects of LRRK1 depletion on Erk phosphorylation. The effects of LRRK1 depletion on phosphorylation of EGFR (compared with total EGFR) should also be shown. This is important because, although accumulation of phosphorylated EGFR upon LRRK1 depletion is shown in Figure 5, this is not quantified and the role of LRRK1 in regulating PTP1B-mediated EGFR dephosphorylation is central to the paper.

Fig 5B shows that when LRRK1 or PTP1B are depleted there are more punctae costaining for GFP and phospho-EGFR suggesting that phosphorylated EGFR is accumulating at membrane contact sites. Whilst this is a very elegant assay, only single cells are shown. Could the number of GFP/phospho-EGFR positive punctae/cell under the different conditions be quantitated?

Figure 6 B is investigating the effect of siLRRK1 on EGFR:PTP1B interaction using a catalytically inactive form of PTP1B which prolongs what is normally a very transient interaction between the phosphatase and its substrate. The authors state that the interaction between PTP1B was dependent on EGFR stimulation and reduced by LRRK2 depletion. However, I can see a band at approximately the right molecular weight in all lanes where EGFR has been transfected. I can see a higher molecular weight band in the control siRNA cells treated with EGF that has been indicated by an arrow but I think all of the lanes are showing EGFR and the 10 minute EGFR band has reduced mobility due to phosphorylation. The differences between the lanes apart from the mobility, appear quite subtle so I think that some sort of quantitation with statistics is required.

The text at the top of page 13 is somewhat confusing. It states that the nonphosphorylatable form of LRRK1 results in “EGFR accumulation of mixed endosomes in the perinuclear region”. What are ‘mixed endosomes’? Also, “These findings suggest that LRRK1 kinase activity remains inactive until the completion of EGFR sorting and requires activation to initiate the endosomal trafficking of EGFR.” What is meant by ‘initiation of endosomal trafficking’? It might be clearer to say ‘transport to the perinuclear region’.

Figure 7 nicely shows that LRRK1 can be dephosphorylated by PTP1B in vitro and that overexpression of a cytosolic form of PTP1B dephosphorylates LRRK1. However the role of endogenous PTP1B in LRRK1 dephosphorylation should be investigated by depleting PTP1B and investigating effects on LRRK1 phosphorylation.

© 2023. Published by The Company of Biologists under the terms of the Creative Commons Attribution License (https://creativecommons.org/licenses/by/4.0/).

That LRRK1 is a substrate of PTP1B is important and this should be included in the abstract, together with sentence summarising the multiple roles of LRRK1 in regulating EGFR traffic.

First revision

Author response to reviewers' comments

Our responses to the comments of Reviewer #1

Major points:

Fig. 6B: There are a couple of concerns about this figure. First, it appears that the amount of phosphorylated EGFR in siLRRK1-treated cells (right lane in the “EGFR, Total lysates” panel) was a bit smaller than that in siControl cells (3rd lane from the right), raising the possibility that no binding of EGFR to Flag-PTP1B(C215S) in siLRRK1-treated cells is due to a smaller amount of phospho-EGFR produced. Regarding this, why was an anti- pY1068-EGFR antibody not used for immunoblotting? Because the anti-EGFR antibody gave fuzzy bands, likely reflecting the formation of phosphorylated forms, use of an anti-pY1068-EGFR antibody may give a clearer result. Use of the latter antibody could also provide direct evidence for sustained activation of EGFR in LRRK1-depleted cells (Fig. 2).

As suggested, we have performed immunoblotting using the anti-pY1068-EGFR antibody. However, in the PTP1B(C215S) immunoprecipitates, we could not detect the clear bands of pY1068-EGFR.

Therefore, we quantified the amount of EGFR immunoprecipitated with PTP1B(C215S) and compared the relative levels of EGF-induced PTP1B-EGFR interaction between control and LRRK1 siRNA-treated cells. We confirm that LRRK1 knockdown decreases the PTP1B-EGFR interaction in response to EGF stimulation (Fig. 6C).

Second, does Flag-PTP1B(C215S) bind to LRRK1 with a similar affinity to Flag- PTB1B wild-type (Fig. 6A)? This should be examined because Flag-PTP1B(C215S) was used for binding experiments (Fig. 6B).

As suggested, we showed that LRRK1 binds to PTP1B(C215S) with a similar affinity to wild-type PTP1B (Fig. 6A).

Fig. 7B: Does pY944-LRRK1 staining disappear for a longer incubation? It is important to show LRRK1 can be dephosphorylated by endogenous PTP1B.

As suggested, we showed that Tyr-944 phosphorylation of LRRK1 disappeared 30 min after EGF stimulation (Fig. 7B). Furthermore, when PTP1B was depleted with siRNA, pY944-LRRK1 signals remained (Fig. 7B). These results confirm that LRRK1 can be dephosphorylated by endogenous PTP1B.

Fig. 7C: Is pY944-LRRK1 present at ER-endosome contact sites? The localization of pY944-LRRK1 seems to be different from that of pY-EGFR (Fig. 5).

We examined whether the pY944-LRRK1 signal was present at the ER-endosome contact sites using the split-GFP system. HeLa S3 cells carrying ERj1(1–200)-V5-GFP_{1-10} and EGFR-GFP_{11} were stimulated with A647-EGF and then immunostained with the anti-pY944-LRRK1 antibody. We showed that several endogenous pY944-LRRK1 signals are localized on the split-GFP foci overlapping with A647-EGF (Fig. S7).

It is better to show that expression of an siRNA-resistant LRRK1 construct can compensate for the phenotype of LRRK1-depleted cells. Alternatively, another siRNA should be tested to exclude the possible off-target effects.

© 2023. Published by The Company of Biologists under the terms of the Creative Commons Attribution License (https://creativecommons.org/licenses/by/4.0/).

As suggested, we examined the effect of another siRNA oligo, LRRK1 siRNA a#1. We confirm that LRRK1 siRNA a#1 also inhibits EGFR sorting into ILVs (Fig. S2).

We have already demonstrated that the expression of siRNA-resistant LRRK1 constructs rescues the defect of EGFR ILV sorting in LRRK1-depleted cells (Hanafusa et al., 2011).

Minor points:

Describe how siRNA-resistant Flag-PTP1B constructs were made.

In this study, we have not used the siRNA-resistant Flag-PTP1B construct.

Fig. 3B-D: How was quadruple staining performed? Because GFP fluorescence overlaps with Alexa-Fluor 488 fluorescence, another fluorescence probe, in addition to Alexa-Fluor 555 and Alexa-Fluor 647, must have been used.

As you pointed out, we described the fluorescence probe Alexa-Fluor 405 in the “MATERIALS and METHODS” section.

Fig. 4A: “ERj(1-220) ---" should read “ERj1(1-220) ---“.

We have corrected this description.

Fig. 5D: How were PTP1BΔC-expressing cells identified? Like Fig. 3B-D, quadruple staining must have been performed.

As suggested, we have performed the quadruple staining (Fig. 5B).

<u>Our responses to the comments of Reviewer #2</u>

Fig. 1A: Endosomes appear larger in the controls than the LRRK1 and PTP1B siRNA- treated cells. Is this the case? If anything I would expect that inhibition of ILV formation would lead to enlarged MVBs. The images should be shown at the same magnification and if the changes in endosome size are consistent then they should be commented upon.

As pointed out, endosomes appeared larger in the controls than in the LRRK1 and PTP1B siRNA-treated cells. In cells depleted of LRRK1 or PTP1B, endosomes were distributed around the plasma membrane (Fig. 1A-C). These results suggest that depletion of LRRK1 or PTP1B may delay fusion between endosomes. We mention this point (p. 8, lines 21–24)

As suggested, we showed the images at the same magnification.

Figure 2 shows the effects of LRRK1 depletion on Erk phosphorylation. The effects of LRRK1 depletion on phosphorylation of EGFR (compared with total EGFR) should also be shown. This is important because, although accumulation of phosphorylated EGFR upon LRRK1 depletion is shown in Figure 5, this is not quantified and the role of LRRK1 in regulating PTP1B-mediated EGFR dephosphorylation is central to the paper.

As suggested, we showed that LRRK1 depletion led to the accumulation of phosphorylated EGFR (Fig. S3).

Fig 5B shows that when LRRK1 or PTP1B are depleted there are more punctae costaining for GFP and phospho-EGFR suggesting that phosphorylated EGFR is accumulating at membrane contact sites. Whilst this is a very elegant assay, only single cells are shown. Could the number of GFP/phospho-EGFR positive punctae/cell under the different conditions be quantitated?

As suggested, we showed quantification of the number of GFP puncta co-localized with pY1068-EGFR (Fig. 5C).

© 2023. Published by The Company of Biologists under the terms of the Creative Commons Attribution License (https://creativecommons.org/licenses/by/4.0/).

Figure 6 B is investigating the effect of siLRRK1 on EGFR:PTP1B interaction using a catalytically inactive form of PTP1B which prolongs what is normally a very transient interaction between the phosphatase and its substrate. The authors state that the interaction between PTP1B was dependent on EGFR stimulation and reduced by LRRK2 depletion. However, I can see a band at approximately the right molecular weight in all lanes where EGFR has been transfected. I can see a higher molecular weight band in the control siRNA cells treated with EGF that has been indicated by an arrow but I think all of the lanes are showing EGFR and the 10 minute EGFR band has reduced mobility due to phosphorylation. The differences between the lanes, apart from the mobility, appear quite subtle so I think that some sort of quantitation with statistics is required.

As suggested, we quantified the amount of EGFR immunoprecipitated with PTP1B(C215S) and compared the relative levels of EGF-induced PTP1B-EGFR interaction between control and LRRK1 siRNA-treated cells. We confirm that LRRK1 knockdown decreases the PTP1B-EGFR interaction in response to EGF stimulation (Fig. 6C).

The text at the top of page 13 is somewhat confusing. It states that the nonphosphorylatable form of LRRK1 results in "EGFR accumulation of mixed endosomes in the perinuclear region". What are 'mixed endosomes'? Also, "These findings suggest that LRRK1 kinase activity remains inactive until the completion of EGFR sorting and requires activation to initiate the endosomal trafficking of EGFR."
What is meant by 'initiation of endosomal trafficking'? It might be clearer to say 'transport to the perinuclear region'.

We have corrected the description.

Figure 7 nicely shows that LRRK1 can be dephosphorylated by PTP1B in vitro and that overexpression of a cytosolic form of PTP1B dephosphorylates LRRK1. However, the role of endogenous PTP1B in LRRK1 dephosphorylation should be investigated by depleting PTP1B and investigating effects on LRRK1 phosphorylation.

As suggested, we showed that the Tyr-944 phosphorylation of LRRK1 disappeared 30 min after EGF stimulation and that when PTP1B was depleted with siRNA, the pY944-LRRK1 signal remained (Fig. 7B).

That LRRK1 is a substrate of PTP1B is important and this should be included in the abstract, together with sentence summarising the multiple roles of LRRK1 in regulating EGFR traffic.

As suggested, we have rewritten the abstract.

Second decision letter

MS ID#: JOCES/2022/260566

MS TITLE: LRRK1 functions as a scaffold for PTP1B-mediated EGFR sorting into ILVs at the ER—endosome contact site

AUTHORS: Hiroshi Hanafusa, Keitaro Fujita, Misa Kamio, Shiori Iida, Yasushi Tamura, Naoki Hisamoto, and Kunihiro Matsumoto
ARTICLE TYPE: Research Article

I am happy to tell you that your manuscript has been accepted for publication in Journal of Cell Science, pending standard ethics checks. As reviewer #1 has some comments about the abstract, please change it in proofs.

© 2023. Published by The Company of Biologists under the terms of the Creative Commons Attribution License (https://creativecommons.org/licenses/by/4.0/).

Reviewer 1

Advance summary and potential significance to field

In the present study Hanafusa and colleagues demonstrated that LRRK1 is required for EGFR dephosphorylation by ER-localized PTP1B, which occurs at ER-endosome contact sites, followed by sorting of dephosphorylated/inactivated EGFR into the intraluminal vesicles of endosomes. LRRK1 does not participate in EGF-induced formation of the ER–endosome contact sites, but does function as a scaffold that mediates the interaction of PTP1B with phosphorylated EGFR, leading to down-regulation of EGF signaling.

This is a well-written paper and provides a new insight into the function of LRRK1, and in the revised version the authors have adequately addressed my concerns.

Comments for the author

I have a couple of minor comments to the abstract.
Line 8 in the abstract: It may be better to state “Active, phosphorylated EGFR”, not to merely state “Active EGFR”.

3rd line from the bottom in the abstract: “the transport of EGFR-containing endosomes” should be changed to “the transport of EGFR-containing endosomes to the perinuclear region”

Reviewer 2

Advance summary and potential significance to field

In this paper the authors build on their previous observations (confirmed in the current study) that LRRK1 regulates the sorting of EGFR onto intraluminal vesicles (ILVs) of MVBs. They convincingly show that LRRK1 regulates the PTP1B-mediated EGFR dephosphorylation that occurs at membrane contact sites between EGFR-containing endosomes and the ER. They further show that LRRK1 is not required for contact site formation using a very elegant split GFP assay that they have validated using a known membrane contact site regulator, annexin 1. They then present evidence that LRRK1 acts as a scaffold to enable EGFR:PTP1B interaction.

One of the most interesting parts of this study is the idea that LRRK1 has 2 roles in EGFR trafficking that are regulated by its phosphorylation. They had previously shown that EGFR phosphorylates LRRK1 on a site that inhibits LRRK1 kinase activity and that LRRK1 kinase activity though not involved in sorting of EGFR onto ILVs is required for movement of EGFR-containing endosomes to the perinuclear region. Here they show that LRRK1 is a substrate of PTP1B. A potential order of events is presented whereby EGFR phosphorylates and inactivates LRRK1 and LRRK1 acts as a scaffold at membrane contact sites to allow PTP1B and EGFR to interact and drive sorting of EGFR onto ILVs. PTP1B then dephosphorylates LRRK1 allowing the LRRk1 kinase activity to promote movement of EGFR containing endosomes to the perinuclear region. This is a very interesting and novel model and would definitely be of potential interest to readers of J Cell Science.

Comments for the author

The authors have satisfied by concerns.

© 2023. Published by The Company of Biologists under the terms of the Creative Commons Attribution License (https://creativecommons.org/licenses/by/4.0/).
